# In-Depth Characterization of *greenflesh* Tomato Mutants Obtained by CRISPR/Cas9 Editing: A Case Study With Implications for Breeding and Regulation

**DOI:** 10.3389/fpls.2022.936089

**Published:** 2022-07-11

**Authors:** Silvia Gianoglio, Cinzia Comino, Andrea Moglia, Alberto Acquadro, Víctor García-Carpintero, Gianfranco Diretto, Filippo Sevi, José Luis Rambla, Gabriella Dono, Danila Valentino, Elena Moreno-Giménez, Mateu Fullana-Pericàs, Miguel A. Conesa, Jeroni Galmés, Sergio Lanteri, Andrea Mazzucato, Diego Orzáez, Antonio Granell

**Affiliations:** ^1^Departamento de Biotecnología de Cultivos, Instituto de Biología Molecular y Celular de Plantas (IBMCP), Consejo Superior de Investigaciones Científicas (CSIC) – Universitat Politécnica de Valéncia (UPV), Valencia, Spain; ^2^Dipartimento di Scienze Agrarie, Forestali e Alimentari (DISAFA), Plant Genetics and Breeding, University of Turin, Turin, Italy; ^3^Italian Agency for New Technologies, Energy and Sustainable Development (ENEA), Rome, Italy; ^4^Department of Agricultural Sciences, University of Naples Federico II, Naples, Italy; ^5^Department of Biology, Biochemistry and Natural Sciences, Universitat Jaume I, Castellón de la Plana, Spain; ^6^Department of Agriculture and Forest Sciences (DAFNE), Università degli Studi della Tuscia, Viterbo, Italy; ^7^Departamento de Biotecnología, Instituto de Agroquímica y Tecnología de los Alimentos (IATA-CSIC), Paterna, Spain; ^8^Instituto de Investigaciones Agroambientales y de Economía del Agua (INAGEA), Research Group on Plant Biology Under Mediterranean Conditions, Universitat de les Illes Balears, Palma, Spain

**Keywords:** CRISPR-Cas9, tomato, staygreen, *greenflesh*, nutritional quality, pathogen resistance, breeding

## Abstract

Gene editing has already proved itself as an invaluable tool for the generation of mutants for crop breeding, yet its ultimate impact on agriculture will depend on how crops generated by gene editing technologies are regulated, and on our ability to characterize the impact of mutations on plant phenotype. A starting operational strategy for evaluating gene editing-based approaches to plant breeding might consist of assessing the effect of the induced mutations in a crop- and locus-specific manner: this involves the analysis of editing efficiency in different cultivars of a crop, the assessment of potential off-target mutations, and a phenotypic evaluation of edited lines carrying different mutated alleles. Here, we targeted the *GREENFLESH* (*GF*) locus in two tomato cultivars (‘MoneyMaker’ and ‘San Marzano’) and evaluated the efficiency, specificity and mutation patterns associated with CRISPR/Cas9 activity for this gene. The *GF* locus encodes a Mg-dechelatase responsible for initiating chlorophyll degradation; in *gf* mutants, ripe fruits accumulate both carotenoids and chlorophylls. Phenotypic evaluations were conducted on two transgene-free T_2_ ‘MoneyMaker’ *gf* lines with different mutant alleles (a small insertion of 1 nucleotide and a larger deletion of 123 bp). Both lines, in addition to reduced chlorophyll degradation, showed a notable increase in carotenoid and tocopherol levels during fruit ripening. Infection of *gf* leaves and fruits with *Botrytis cinerea* resulted in a significant reduction of infected area and pathogen proliferation compared to the wild type (WT). Our data indicates that the CRISPR/Cas9-mediated mutation of the *GF* locus in tomato is efficient, specific and reproducible and that the resulting phenotype is robust and consistent with previously characterized *greenflesh* mutants obtained with different breeding techniques, while also shedding light on novel traits such as vitamin E overaccumulation and pathogen resistance. This makes *GF* an appealing target for breeding tomato cultivars with improved features for cultivation, as well as consumer appreciation and health.

## Introduction

CRISPR-mediated gene editing has opened exciting perspectives for the development of sustainable, innovative crops with improved agronomic, nutritional quality and industrial processing traits (Zhang et al., 2020; Zhu et al., 2020; Maximiano et al., 2021). Since its inception, the array of gene editing tools has been steadily expanding, thanks to the diversification of its applications and the increasing number of endonucleases available to target specific sequence patterns (Collias and Beisel, 2021). However, to date, protocols for routine application of such technologies and in-depth evaluations of gene editing efficiency are available only for some plant species for which extensive genomic resources and efficient transformation and regeneration methods are available.^1^

While gene editing has already proved itself as an invaluable tool for generating mutants for research applications, its ultimate impact on agriculture and the food supply chain will inevitably depend on whether and how countries will choose to regulate crops generated by employing this technology. Regulatory issues are multi-layered, and can be briefly summarized as follows: (i) whether gene edited crops need to be specifically regulated; (ii) if so, whether regulation should be process- or product-triggered; (iii) especially for process-triggered regulatory systems, whether existing regulation on genetically modified crops is applied, or a new framework should be put in place. The different stances legislators and experts have taken on these matters have been the subject of extensive reviews (Eckerstorfer et al., 2019a,b; Friedrichs et al., 2019; Lassoued et al., 2021). The type of mutation introduced in a crop as well as the technology used to obtain it are decisive in guiding the legislator toward a normative decision. Mutations induced by site-directed nucleases have been classified in different categories, depending on whether the initial DNA breaks are repaired randomly by Non-Homologous End Joining (NHEJ, SDN-1 mutations), by Homologous Recombination (HR) followed by small template-directed modifications (SDN-2) or by HR followed by the insertion of foreign DNA fragments (SDN-3). However, the structural characteristics of a mutation are hardly predictive of its phenotypic effects, since single base indels or substitutions can have major phenotypic consequences, while more extensive rearrangements may not result in correspondingly greater alterations. A consideration which is often made for bypassing or simplifying the regulation for gene-edited crops is whether the induced mutation mimics alleles already present in the genetic pool of a crop or in its wild relatives (Rostoks, 2021).

However, a precise recapitulation at the nucleotide level of natural mutant alleles is technically difficult, as it often requires a HR (SDN-2) approach, which currently is not available for most crops. The routine gene-editing approach consists of inducing at target loci random mutations that are functionally equivalent to natural ones, although the functional equivalence of induced and natural mutations is not always easy to predict. For instance, CRISPR-induced missense mutations in tomato ripening genes such as *RIN* and *NOR* failed to fully recapitulate the late ripening phenotype of the natural mutants, allegedly because such alleles produce earlier gain-of-function truncated forms of the protein (Ito et al., 2017; Gao et al., 2019, 2020). This raises the question of whether these mutations actually result in gene knockouts or rather in a shift of activity or transcriptional regulation.

Irrespective of general considerations on whether SDN-1 approaches should be deregulated *per se*, a starting operational strategy would consist of establishing the suitability of a SDN-1 approach in a crop- and locus-specific manner. Such assessment involves the analysis of the efficiency of a particular SDN-1 approach in different cultivars of a crop, the assessment of potential off-target mutations, and a phenotypic analysis of different SDN-1-induced alleles within the same cultivar to identify possible allele-specific phenotypes. However, there are few examples in literature in which the safety and robustness of a modification induced by a SDN-1 mutation have been extensively studied in a locus- and crop-specific manner. One such example is that of the tomato tangerine (*t*) locus, which corresponds to the carotenoid isomerase (*CRTISO*) gene involved in lycopene synthesis (Dahan-Meir et al., 2018; Ben Shlush et al., 2021; Jayaraj et al., 2021).

In this paper, we focused on the *GF* (*GREEN FLESH*/*STAYGREEN*) locus in tomato, which encodes a Mg dechelatase required for chlorophyll catabolism (Shimoda et al., 2016). We used CRISPR/Cas9 to knock out the *GF* locus in two tomato cultivars (‘MoneyMaker’ and ‘San Marzano’) and evaluated the efficiency and robustness of this approach. These two cultivars were selected as examples of widely used and highly productive tomato varieties for fresh consumption and industrial processing, which also have different fruit morphologies. Moreover, ‘MoneyMaker’ is routinely used as a model cultivar for research applications. The *GF* locus was chosen for both its biological significance and its genetic characteristics. It belongs to the group of so-called ‘staygreen’ mutants, which comprises a class of phenotypes with impaired or delayed chlorophyll catabolism. In some of these phenotypes, called functional staygreen, senescence and ripening are halted or delayed, while in others, called cosmetic staygreen, senescence and ripening mostly proceed as in wild types, but vegetative and reproductive organs display a characteristic phenotype caused by chlorophyll retention (Thomas and Ougham, 2014). In the case of *gf* tomatoes, which belong to the latter group, not only do senescent leaves have extremely delayed or no yellowing, but most distinctively ripe fruits also develop a characteristic color due to the simultaneous presence of chloroplasts and chromoplasts/plastoglobules (Barry et al., 2008). A number of such tomato varieties, referred to as brown, black or “chocolate,” are commercially available and are highly appreciated by consumers; they carry different mutations at the same locus (deletions ranging in size from 2 to 1,163 bp, single-base substitutions and single-base insertions) and all result in comparable phenotypes (Barry and Pandey, 2009). Despite not dramatically altering senescence and ripening, the *gf* mutation does result in some potentially useful functional changes mainly related to carotenoid accumulation and plastid morphology (Roca et al., 2006; Grassl et al., 2012; Luo et al., 2013; Zhu et al., 2021). In this work, we performed phenotypic evaluations on the Cas9-free T_2_ generation of two ‘MoneyMaker’ *gf* lines, one carrying single nucleotide insertion and the other a 123 bp deletion at the *GF* target locus. Fruit metabolic profiles were assessed, with emphasis on compounds relevant for health and aroma, to better define whether the phenotypic changes are just “cosmetic.” In other plant species, mutation of *GF* orthologs has been associated with tolerance against bacteria in Arabidopsis (Mecey et al., 2011) and fungal pathogens in *Medicago truncatula* (Ishiga et al., 2015), cucumber (Pan et al., 2018; Wang et al., 2019) and soybean (Chang et al., 2019). We also explored the link between the tomato *gf* mutation and pathogen tolerance by infecting leaves and fruits with the gray mold *B. cinerea* and assessing symptoms and pathogen growth, finding reduced pathogen growth in *gf* mutants. In summary, our results point to considerable efficiency and reproducibility when generating *gf* mutants through CRISPR/Cas9. The two mutant lines we assessed, despite carrying structurally different alleles, are phenotypically equivalent and closely recapitulate known features of tomato *gf* mutants obtained with traditional breeding techniques or with other genetic engineering technologies. In addition, we were able to establish the previously undescribed relationship existing in tomato between these mutants and pathogen resistance.

## Materials and Methods

### Golden Braid-Assisted Construction of the CRISPR/Cas9 Vector

The pDGB3α2 Tnos:nptII:Pnos—U6-26:gRNA:scaffold—P35S:hCas9:Tnos (GB4587) vector was built following the standard GoldenBraid assembly strategy as described by Vázquez-Vilar et al. (2021). The gRNA targeting the *GF* tomato locus (Solyc08g080090) was designed using the Benchling CRISPR tool^2^ and it was selected to target the third exon. The level 1 GB construct pDGB3α1 U6-26:gRNA:scaffold was confirmed by restriction enzyme (RE) analysis and sequencing; level >1 constructs, which incorporated the *hCas9* and *nptII* transcriptional units, were obtained through bipartite *Bsm*BI- or *Bsa*I-mediated reactions and were checked by RE-analysis. All GB parts are available at http://gbcloning.upv.es under the correspondent GB database ID, and their names and sequences are reported in Supplementary Table 1. The vector was finally transferred to *A. tumefaciens* LBA4404 electrocompetent cells.

### Tomato Stable Transformation

Stable transformation of ‘MoneyMaker’ and ‘San Marzano’ was performed according to the protocol reported by Ellul et al. (2003), with minor modifications. *In vitro* culture was performed in a long day growth chamber (16:8 light:dark cycle) at 24°C, 60–70% humidity and 250 μmol photons m^–2^ s^–1^. For initial assessment of editing events, 150 mg of leaf tissue were collected from three different leaves of *in vitro* fully grown, rooted plantlets, frozen in liquid nitrogen and stored at −80°C until DNA extraction. For ‘MoneyMaker,’ a second editing assessment was performed on a selected pool of genotypes growing in the greenhouse, using 150 mg of plant material from two leaves and two fruits from each plant.

### Evaluation of Mutation Frequencies at On-Target and Off-Target Sites

Genomic DNA was extracted according to a CTAB protocol (Murray and Thompson, 1980) and treated with RNAse A to remove RNA contamination. DNA quality was checked by 0.8% (w/v) agarose gel electrophoresis and quantification was carried out with Qubit 2.0 (Life Technologies, Carlsbad, CA, United States) based on the Qubit dsDNA HS Assay (Life Science).

Mutation frequencies at the target and off-target sites were evaluated according to an adapted version of the 16S Metagenomic Sequencing Library preparation protocol provided by Illumina (16S Sample Preparation Guide: https://support.illumina.com/documents/documentation/chemistry_documentation/16s/16s-metagenomic-library-prep-guide-15044223-b.pdf). Putative off-target sites were selected using CasOFFinder^3^ as representatives of the different possible configuration and position of mismatches relative to the PAM. Their characteristics are listed in Supplementary Table 2. DNA amplifications were carried out using the KAPA HiFi HotStart ReadyMix PCR Kit (Kapa Biosystems, Boston, MA, United States). Dual indexing was done using the Nextera XT system (Illumina, San Diego, CA, United States) using 16 i5 indexes (S502-S522) and 24 i7 indexes (N701-N729), enabling the multiplexing of 384 individual libraries. DNA sequencing was performed with an Illumina MiSeq sequencer (Illumina Inc., San Diego, CA, United States) and 150 bp paired-end reads were generated. After sequencing, adapter sequences were removed and reads less than 50 nucleotides long were discarded using Trimmomatic v0.39 (Bolger et al., 2014). Processed reads were analyzed for possible CRISPR-Cas9 editing events with CRISPResso2 (Clement et al., 2019), with -min_paired_end_reads_overlap to 1, leaving the other options by default.

A selected pool of T_0_ ‘MoneyMaker’ plants underwent a second genetic screening 3 months after having been transferred to the greenhouse, sampling two leaves and two fruits per plant: their genotypes were evaluated through Sanger sequencing and chromatogram decomposition analysis using TIDE (TIDE | Web tool: https://tide.nki.nl/). The T_1_ and T_2_ selected progenies were screened throughS anger sequencing for *gf* genotyping and through Illumina Amplicon Sequencing at putative off-target loci. All oligonucleotide and primer sequences used in this work are listed in Supplementary Table 3.

### Selection of the Edited, Transgene-Free ‘MoneyMaker’ Progeny

In the T_0_ generation, five edited events of the ‘MoneyMaker’ variety were chosen, grown in the greenhouse and their seeds collected to identify, through amplification of the *Cas9* and *nptII* genes, T_1_ individuals in which the T-DNA had been segregated from the mutated *gf* locus. Five transgene-free edited individuals were selected; among these, two lines (2B19 and 12A41) were selected as élite for being homozygous for *gf* alleles of interest, and their T_2_ progeny was phenotypically evaluated. Their fruits were sampled at six ripening stages, from mature green (MG) to breaker (Br), and every 2 days until 8 days after breaker (Br+2 to Br+8). For each ripening stage and for each line (2B19, 12A41, and WT), three samples were evaluated; each sample consisted of a pool of four fruits. Fruits were collected, snap-frozen in liquid nitrogen and ground using the IKA^®^ A11 basic analytical mill (IKA, Staufen, Germany).

### Extraction and Analysis of Isoprenoids

Extractions of isoprenoids in fruits at six stages of ripening were performed as described previously by Diretto et al. (2020). Briefly, for each sample 5 mg of freeze-dried powder were extracted with chloroform (spiked with 50 mg/l DL-α-tocopherol acetate as internal standard) and methanol (2:1 by volume); 1 volume of 50 mM Tris buffer (pH 7.5, containing 1 M NaCl) was then added and the samples were kept for 20 min on ice before a centrifugation step at 15,000 *g* for 10 min at 4°C. The hypophase was collected and the aqueous phase was re-extracted with the same amount of spiked chloroform; the two organic phases were merged and dried by speedvac and the resulting pellets were resuspended in 50 μl of ethyl acetate. For each sample, at least two independent extractions were performed. LC-DAD analyses were carried out using an Accela U-HPLC system (Thermo Fisher Scientific, Waltham, MA, United States). LC separations were performed using a C30 reverse-phase column (100 x 3.0 mm) from YMC (YMC Europe GmbH, Schermbeck, Germany) with mobile phases composed by methanol (A), water–methanol (20:80 by volume) containing 0.2% ammonium acetate (B) and tert–methyl butyl ether (C). The gradient was 95% A : 5% B for 1.3 min, followed by 80% A : 5% B : 15% C for 2.0 min and by a linear gradient to 30% A : 5% B : 65% C over 9.2 min. UV–visible detection was performed continuously from 220 to 700 nm with an online Accela Surveyor photodiode array detector (PDA; Thermo Fisher Scientific). Mass ionization was performed with an atmospheric-pressure chemical ionization (APCI) probe, operating in both + and – voltage conditions. Nitrogen was utilized at 20 and 10 units as sheath and auxiliary gas, respectively. The vaporizer and capillary temperature were set at 300 and 250°C, respectively. The discharge current was 5.5 μA, while S-lens RF level was set at 50. A mass range of 110/1,600 *m/z* was used both in positive and in negative voltage with the following parameters: resolution set at 70,000; microscan, AGC target and maximum injection time equal to, respectively, 1, 1x106 and 50. All solvents used were LC-MS grade quality (CHROMASOLV^®^ from Sigma-Aldrich, Saint Louis, MO, United States). Different isoprenoid classes (carotenoids, chlorophylls, tocochromanols, and quinones) were identified based on the accurate masses and by comparison with authentic standards, when available, and quantified based on the internal standard (IS) amounts (thus named Fold IS); finally, *gf* data were normalized and expressed relatively to WT data (thus reported as Fold WT).

### Extraction and Analysis of Volatile Compounds

The analysis of volatile compounds was performed by means of headspace solid phase microextraction (HS-SPME) coupled to gas chromatography and mass spectrometry (GC/MS) following the protocol reported by Rambla et al. (2017). Roughly, 500 mg of frozen tomato pericarp powder were introduced in a 15 ml glass vial and incubated with a closed cap at 37°C for 10 min in a water bath. Then, 500 ml of an EDTA 100 mM, pH 7.5 solution and 1.1 g of CaCl_2_.2H_2_O were added, mixed gently and sonicated for 5 min. One mL of the resulting paste was transferred to a 10 ml screw cap headspace vial with silicon/PTFE septum and analyzed within 10 h. Volatile compounds in the headspace were extracted by means of a 65 μm PDMS/DVB solid phase microextraction fiber (SUPELCO, Bellefonte, PA, United States). Volatile extraction was performed automatically by means of a CombiPAL autosampler (CTC Analytics, Zwingen, Switzerland). Vials were first incubated at 50°C for 10 min with agitation at 500 rpm. Then, the fiber was exposed to the headspace of the vial for 20 min with the same conditions of agitation and temperature. Volatiles were desorbed at 250°C during 1 min in splitless mode in the injection port of a 6890N gas chromatograph (Agilent, Santa Clara, CA, United States). After desorption, the fiber was cleaned in an SPME fiber conditioning station (CTC Analytics) at 250°C for 5 min with helium flow. Chromatography was performed on a DB-5ms (60 m, 0.25 mm, 1.00 μm) capillary column (J&W, Agilent) with helium as carrier gas at a constant flow of 1.2 ml/min. Temperatures of GC interface and MS source were 260 and 230°C, respectively. Oven programming conditions were 40°C for 2 min, 5°C/min ramp until 250°C and a final hold at 250°C for 5 min. Data was recorded in a 5975B mass spectrometer (Agilent) in the 35–250 *m/z* range at 6.2 scans/s, with 70 eV electronic impact ionization. Chromatograms were processed by means of the Enhanced ChemStation E.02.02 software (Agilent). Compound identification was performed by comparison of both retention time and mass spectrum with those of pure standards. All the standards were purchased from Sigma-Aldrich except 1-nitro-2-phenylethane, which was obtained from Apin Chemicals (Compton, United Kingdom). For quantitation, one specific ion was selected for each compound, and the corresponding peak from the extracted ion chromatogram was integrated. The criteria for ion selection were the highest signal-to-noise ratio and being specific enough to provide good peak integration in that region of the chromatogram. An admixture reference sample was prepared for each season by mixing thoroughly equal amounts of each sample. A 500 mg aliquot of the admixture was analyzed regularly (one admixture for every six to seven samples) and processed as a regular sample as part of the injection series. This admixture contained all the compounds identified in the samples, and it was used as a reference to normalize for temporal variation and fiber aging. Finally, the normalized results (corrected for temporal variation and fiber aging) for a particular sample were expressed as the ratio of the abundance of each compound in that sample to those present in the reference admixture.

### Photosynthetic Activity Assays

For photosynthetic activity assays, seeds of the ‘MoneyMaker’ genotypes 12A26 and 12A50 were sown in 10 l pots containing a substrate mixture of horticultural substrate and perlite (3:1 v/v). For each genotype, four plants were used. All plants were grown in a glass greenhouse in Mallorca during late winter and early spring (mean temperature 25°C, with maximum temperature of 33°C). Plants were irrigated every 2 days to full capacity and with Hoagland’s solution 50% once a week, to avoid growth restrictions related with water or nutrient deficit. Leaf gas-exchange and chlorophyll *a* fluorescence were measured 2 months after seed germination simultaneously with an open infrared gas-exchange analyzer system equipped with a leaf chamber fluorometer (Li-6400–40, Li-Cor Inc., United States). Measurements were performed in two types of leaves: (1) young fully expanded leaves, located in the upper part of the plant, and (2) basal leaves, being among the oldest but not presenting chlorotic or necrotic symptoms. Along the measurements, the vapor pressure deficit (VPD) ranged between 1.1 and 2.8 kPa, with a mean of 2.1 kPa. Measurements were performed from 09:00 to 12:00. Environmental conditions in the leaf chamber consisted of a photosynthetic photon flux density of 1,500 μmol m^–2^ s^–1^ (with 10% blue light), and a leaf temperature of 25°C. Measurements were performed after inducing steady-state photosynthesis for at least 5 min at an ambient CO_2_ concentration (C_a_) of 400 μmol CO_2_ m^–2^ s^–1^. The quantum efficiency of the photosystem II (PSII)-driven electron transport was determined using Equation (1):


(1) Φ⁢P⁢S⁢I⁢I=F′M-FsF′M

where F_s_ is the steady-state fluorescence in the light (PPFD 1,500 μmol photon m^–2^ s^–1^) and F′_M_ the maximum fluorescence obtained with a light-saturating pulse (8,500 μmol photon m^–2^ s^–1^) (Genty et al., 1989). Leaf greenness was measured using a chlorophyll meter (SPAD 502, Konica Minolta, Osaka, Japan) simultaneously with leaf gas exchange measurements in the same leaves.

### Pathogen Susceptibility Assays

The susceptibility of tomato fruits to *B. cinerea* was assayed by inoculating ripe fruits (Br+8) of the 2B19 and 12A41 T_2_ lines, together with WT tomatoes, with 10 μl of a suspension of 8 × 10^5^ conidia ml^–1^, through a pipette tip. Two inoculations were performed for each fruit at two diametrically opposed spots around the equator. Six fruits were inoculated for the WT, six for line 2B19 and five for line 12A41. Infection area was measured 7 days post infection (dpi) with graph paper. Leaf susceptibility assays were performed by infecting the detached 5th and 6th compound leaves of 5-week-old plants of the 2B19 and 12A41 T_2_ lines, together with WT leaves, with 5 μl of a suspension of 10^6^ *B. cinerea* conidia ml^–1^, in four distant spots per leaf. For each genotype, six leaflets were infected. Prior to infection, leaves were rinsed in sterile distilled water and, once infected, they were kept in Petri dishes in a high-humidity chamber. The progression of the infection was checked daily, and 7 dpi the necrotic area around the infection spot was measured using ImageJ (Schneider et al., 2012). Comparable amounts of leaf tissue in the area included between infection spots was collected 7 dpi. Genomic DNA was extracted using the protocol reported by Khang et al. (2007). The abundance of pathogen DNA was estimated in three samples per genotype through qPCR by amplifying the *B. cinerea* intergenic spacer (IGS) as reported by Suarez et al. (2005).

### Statistical Analysis

IBM SPSS Statistics for Windows, version 25 (IBM Corp., Armonk, NY, United States) was used to perform univariate analyses. Multivariate statistical analyses were performed with MetaboAnalyst 5.0^4^ and the SIMCA-P version 13.0 software (Umetrics, Malmö, Sweden).

## Results

### Target Selection and Vector Assembly

**FIGURE 1:**
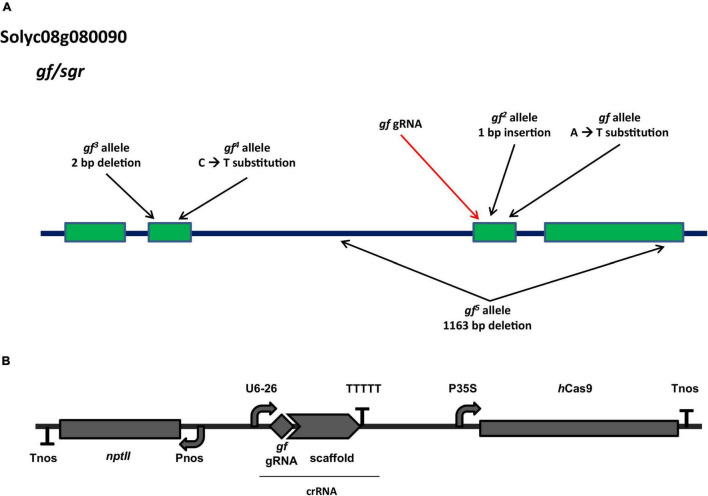
Characteristics of the *GF* locus and of the gene editing construct. **(A)** Architecture of the Solyc08g080090 (*greenflesh*) locus and known natural mutations. The position of known mutations present in heirloom tomato varieties is indicated by black arrows. The red arrow indicates the position targeted by the *gf* gRNA used in this study. Adapted from Barry and Pandey (2009). **(B)** Architecture of the T-DNA used to generate tomato edited *gf* mutants. Expression of the Cas9 endonuclease is controlled by the CaMV 35S promoter and the nopaline synthase (nos) terminator; gRNA expression is driven by the Arabidopsis PolIII U6-26 promoter; the *nptII* selection marker for kanamycin resistance is expressed under the control of the nos promoter.

Despite not having annotated functional domains, GF proteins possess a highly conserved N-terminal region of about 150 amino acids, which targets the protein to the chloroplast, a highly conserved central core and a conserved cysteine-rich motif in the otherwise variable C-terminal region. A gRNA was selected to target the third exon, with the Cas9 cut site at position 1749, in correspondence with the conserved central core and in proximity to the known *gf* ^2^ (pos. 1768) and *gf* (pos. 1789) alleles (Figure 1A). The gene editing module carrying the human codon optimized Sp*Cas9*, the gRNA and the selection marker (*nptII*) transcriptional units (Figure 1B) was assembled according to the GB 4.0 syntax. Our strategy was directed at producing loss of function mutations similar to the spontaneous mutations characterized for the *GF* locus (Barry and Pandey, 2009), which are all predicted to result in truncated forms of the GF protein, with similar effects on mutant visual phenotypes.

### Editing of the *GF* Locus Is Efficient and Robust in Two Tomato Cultivars

**FIGURE 2:**
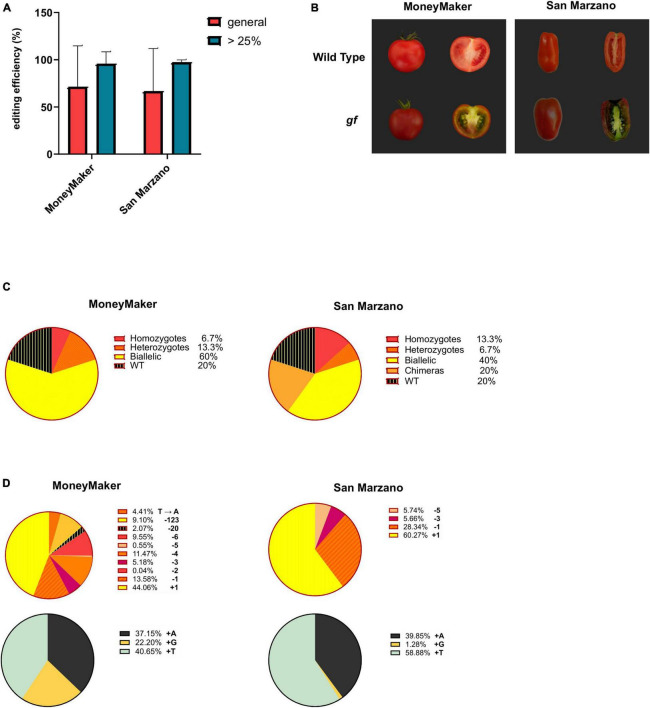
Evaluation of editing events. **(A)** Editing efficiency for the ‘MoneyMaker’ and ‘San Marzano’ tomato varieties. Editing efficiency for each cultivar is expressed as the means of the editing efficiencies for each plant, these being calculated as the % of mutated reads for each individual. Red bars indicate the editing efficiency for the general ‘MoneyMaker’ or ‘San Marzano’ population (15 plants each), while blue bars represent the editing efficiency in the sub-population of plants with editing levels above 25%. Error bars indicate standard deviations. Editing efficiencies between cultivars are not significantly different (Student’s *t*-test, *p* = 0.77 for the general population, *p* = 0.7 for the sub-population with editing above 25%). **(B)** *gf* phenotype in ripe fruits of the ‘MoneyMaker’ and ‘San Marzano’ cultivars. Ripe fruits of the two cultivars share a similar phenotype, with darkened pericarp and mesocarp due to the simultaneous presence of chlorophylls and carotenoids, and green placenta. **(C)** Allelic configuration of the ‘MoneyMaker’ and ‘San Marzano’ *gf* populations. Pie charts represent the frequency of each allelic configuration in the two cultivars (WT, chimeric, heterozygous, biallelic, or homozygous). **(D)** Allelic frequencies in the ‘MoneyMaker’ and ‘San Marzano’ *gf* populations. The upper pie charts represent the frequency of each detected allele in ‘MoneyMaker’ and ‘San Marzano’ edited T_0_ plants, while the lower pie charts detail the frequency and identity of single nucleotide insertions, the most common mutations found in both cultivars.

For each cultivar (‘MoneyMaker’ and ‘San Marzano’), 15 independent T_0_ plants regenerated on selective media were analyzed. Genotyping was performed through high-throughput Illumina amplicon sequencing of the target locus. Transgene integration was confirmed for all analyzed plants by PCR amplification of the *Cas9* gene on tomato genomic DNA. The average gene editing efficiency was 71.38% for ‘MoneyMaker’ and 66.66% for ‘San Marzano’ (Figure 2A). Editing levels for individual mutants are reported in Table 1. It is worth noting that in most cases the observed differences were between plants in which gene editing had occurred with very high efficiency and those in which it had occurred at very low rates or not at all, pointing to a divergence in transgene expression rather than in efficiency of the editing machinery. Excluding plants with editing levels below 25% (from which it would be less likely to retrieve an edited progeny), i.e., four plants in the ‘MoneyMaker’ dataset and five for ‘San Marzano,’ efficiencies were consistently similar (‘MoneyMaker’ 89%, ‘San Marzano’ 90.66%) and standard deviations were greatly reduced (Figure 2A). Differences in editing efficiency between the two cultivars were not significant either in the general population, or in the subset of plants with over 25% editing. The visual phenotype of ripe fruits also showed similar traits between the two cultivars (Figure 2B). Chlorophyll retention was visible in all fruit tissues and in the green coloration of the placenta.

**TABLE 1:** Editing efficiencies for the *GF* target.

MoneyMaker		San Marzano	
	
Sample	Efficiency	Sample	Efficiency
MM1	100.00	SM1	97.98
MM2	0.00	SM2	99.33
MM3	57.42	SM3	0.84
MM4	100.00	SM4	97.78
MM5	100.00	SM5	90.89
MM6	100.00	SM6	1.80
MM7	100.00	SM7	0.00
MM8	100.00	SM8	96.49
MM9	0.48	SM9	0.00
MM10	0.00	SM10	96.22
MM11	97.30	SM11	100.00
MM12	17.43	SM12	97.88
MM13	100.00	SM13	24.19
MM14	100.00	SM14	98.94
MM15	98.03	SM15	97.53
MM WT	0.31	SM WT	1.46

*Efficiency is calculated as % of edited reads generated for each plant.*

The most frequent allelic configuration in edited plants was biallelic (with two different edited alleles) both for ‘MoneyMaker’ (60%) and ‘San Marzano’ (40%) (Figure 2C). 20% of ‘San Marzano’ mutants were chimeric, contrary to the ‘MoneyMaker’ population, where no chimeras were present. The percentage of WT, unedited individuals was 20% for both cultivars. Edited alleles ranged in size from an insertion or deletion of 1 nucleotide to a 123 bp deletion (Figure 2D). Ten different alleles were found in ‘MoneyMaker’ plants and six in ‘San Marzano.’ The most common mutation was a single nucleotide insertion (44% for ‘MoneyMaker,’ 60% for ‘San Marzano’) and, specifically, the insertion of a thymine represented 18% of total edited alleles in ‘MoneyMaker’ and 35% in ‘San Marzano,’ making it the most common mutation of our gene editing at the targeted *GF* locus (29% of the total edited alleles found in the two cultivars, combined). Small deletions (<10 bp) represented the second most frequent editing outcome (41% in ‘MoneyMaker,’ 40% in ‘San Marzano’). Larger deletions were found at lower frequencies: in ‘MoneyMaker,’ a 20 bp and a 123 bp deletions in, respectively, 2 and 9% of the total edited alleles. In ‘San Marzano,’ no deletions larger than 5 bp were found. Also, a single base substitution (T → A) was found in ‘MoneyMaker’ (4%). For the ‘MoneyMaker’ cultivar, three edited lines (2B, 3C, and 10B, corresponding to MM3, MM6, and MM11 in Table 1, respectively) were subjected to a second molecular screening 3 months after having been transferred to the greenhouse, with the aim to monitor Cas9 activity across plant growth and development, sampling two leaves and two fruits per plant (Supplementary Figure 1). For lines 3C and 10B, which already carried only mutated alleles, no further changes were detected at the target locus. In line 2B, which initially showed a 57.42% editing efficiency, Cas9 activity increased during plant growth, reaching almost complete editing in some of the sampled tissues (96.3% in one of the analyzed fruits). This indicates that, where a WT allele is still present, Cas9 can keep on introducing changes. While no new alleles were introduced, this additional activity span resulted in an enrichment of the +G and +T alleles already detected in plant 2B.

### No Gene Editing Activity Was Detectable at Putative Off-Target Sites

**FIGURE 3:**
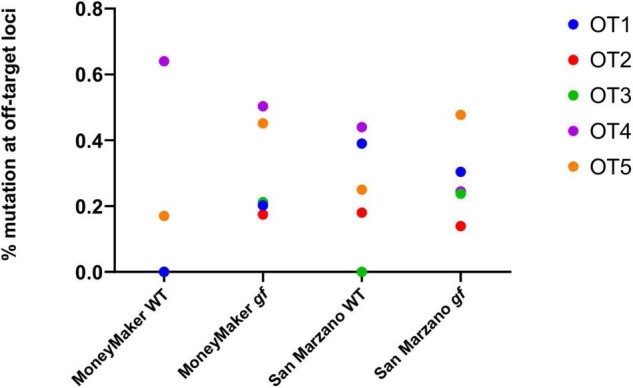
Frequencies of mutation at putative off-target loci. Five putative off-target loci were analyzed for CRISPR-induced mutations. For each population and each putative off-target (OT), variation is expressed as the means of mutation rates for each plant, these being calculated as the % of mutated reads for each individual. All detected mutations are SNPs, no indels were reported. The combined mutation rates for all five OTs were compared between wild-type and edited plants, and no significant differences were found (ANOVA, *p* = 0.66).

Five putative off-target sequences were selected among those identified by CasOFFinder, as representatives of different kinds and numbers of mismatches. Their characteristics are listed in Supplementary Table 2. In general, no sequences can be found in the tomato genome with less than three mismatches with respect to the *gf* gRNA used in this study. Among the selected putative off-targets, three correspond to non-coding regions of the tomato genome, while two correspond to annotated genes. Targeted deep sequencing at these loci proved that indeed no unspecific editing was detectable, confirming existing data on the high specificity of Cas9-mediated gene editing in plants (Figure 3; Maioli et al., 2020). To understand the general impact of off-target effects, for each cultivar the total variation at putative off-target sites was analyzed. Mutation rates for all five off-targets were consistently below 1% in both cultivars, and the rare detectable mutations were not indels, but substitutions, likely due to SNPs or base misattributions. Notably, no significant differences were found between edited and WT plants for these loci.

### Segregation of the Transgene and of Mutated Alleles in the ‘MoneyMaker’ T1 and T2 Progeny

Two ‘MoneyMaker’ plants (2B and 12A, MM3 and MM13 in Table 1, respectively) were selected for further characterization. Both T_0_ plants were biallelic: MM3 carried two different single-base insertions (+T and +G), while MM13 carried a 4 bp deletion and the larger deletion found in the dataset (123 bp). Segregant, transgene-free T_1_ progenies were selected for both lines through amplification of the *Cas9* and *nptII* genes and stable inheritance of the edited *gf* alleles was confirmed by PCR and Sanger sequencing. T_1_ plants 2B19 and 12A41 were selected as transgene-free, homozygous lines to yield a T_2_ progeny used for in-depth metabolic characterization and for pathogen susceptibility assays. Line 2B19 carries a single base insertion (+T), while line 12A41 carries a 123 bp deletion. In addition to genotyping the *GF* locus, T_1_plants and their progenies were also screened at the five putative off-target loci identified before: lack of mutations was confirmed, which led us to confidently treat these progenies as isogenic lines suitable for faithful phenotypic characterization. Their genotypes are summarized in Supplementary Table 4.

### Metabolic Profiling of Two Isogenic Mutant Lines of ‘MoneyMaker’ at the *GF* Locus

**FIGURE 4:**
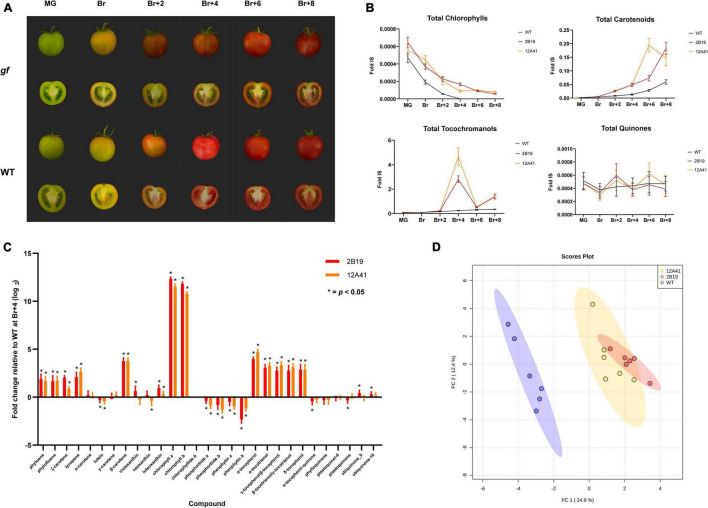
LC-MS analysis of the isoprenoid composition of two isogenic *gf* ‘MoneyMaker’ lines. **(A)** Fruit phenotype during ripening. *gf* and wild-type ‘MoneyMaker’ fruits at six ripening stages: mature green (MG), breaker (Br), and 2, 4, 6, and 8 days after breaker (Br+2 to Br+8). **(B)** Abundance of different isoprenoid classes during ripening. The accumulation trend of four classes of isoprenoid compounds (chlorophylls, carotenoids, tocochromanols, and quinones) was monitored across six ripening stages (MG to Br+8). The abundance of each compound is expressed relative to the internal standard (Fold IS). **(C)** Metabolite composition at Br+4. Relative abundance of a total of 29 isoprenoid compounds in edited *gf* fruits compared to the wild-type, 4 days after breaker. Asterisks signal significant differences between mutant and WT (Student’s *t*-test, *p* < 0.5). **(D)** Principal Component Analysis (PCA) of the metabolite composition of WT and *gf* fruits at Br+4.

The development of tomato fruits of the 2B19 and 12A41 lines was monitored during ripening, from the mature green (MG) stage to eight days after breaker (Br+8). A distinctive phenotype associated with the *gf* mutation, characterized by a green placenta and darker pericarp and mesocarp determined by chlorophyll retention alongside carotenoid accumulation, was clearly visible in fruits starting from breaker (Br) (Figure 4A). The levels of a total of 29 isoprenoid compounds (chlorophylls, carotenoids, tocochromanols, and quinones) were monitored and significant differences were found between *gf* mutants and WT fruits throughout the ripening process for all classes of compounds (Figure 4B and Supplementary Data Files 1, 2).

#### Chlorophylls

As expected, the most dramatic changes were associated with chlorophyll metabolism. More in detail, in *gf* mutants the levels of chlorophyll *a* and *b* and of their catabolites were comparable to the WT at the MG stage. After breaker, chlorophylls decreased with a similar trend in WT and mutant fruits, but the latter were characterized by significantly higher values until Br+8 (Figure 4B). Concurrently, chlorophyll catabolites (chlorophyllide *b*, pheophytin *a* and *b*, and pheophorbide *a* and *b*) were markedly less abundant at all time points, starting from breaker. Both trends are consistent with the role of the GF protein as the Mg dechelatase which converts chlorophyll *a* to pheophytin *a* initiating chlorophyll catabolism (Shimoda et al., 2016).

#### Carotenoids

In addition, total carotenoid content increased in mutant fruits after breaker: indeed, both *gf* lines displayed a 3.7-fold increase in total carotenoid content with respect to WT at Br+4, and line 12A41 further showed a 6.85-fold increase at Br+6. In both edited and WT fruits carotenoid levels continued to increase until Br+8, when 2B19 and 12A41 *gf* lines still displayed 3- and 2.5-fold increase over the WT, respectively (Figure 4B). At the MG stage, the difference in composition between *gf* and WT fruits lied mostly in lycopene content (Supplementary Figure 2). Notably, while carotenoids produced in the early steps of the biosynthetic pathway were enriched in *gf* fruits at all ripening stages, xanthophyll levels did not vary with the same amplitude. In particular, lutein levels slightly increased at Br+2 and Br+6, but had an opposite tendency at Br, Br+4 and Br+8, and violaxanthin, luteoxanthin, and neoxanthin resulted unaltered or were even less abundant in *gf* compared to WT fruits at all ripening stages. At Br+6 and Br+8 carotenoid composition converged in edited and WT lines, while total carotenoid content remained higher in *gf* fruits.

#### Tocochromanols and Quinones

A series of additional changes were observed in other isoprenoid classes: for instance, tocochromanol content was generally enriched in *gf* fruits with an approximate 1.47-fold increase over the WT; however, it peaked at Br+4, with a 11-fold increase in line 2B19 and a 19-fold increase in line 12A41, and again less prominently at Br+8, with a 4-fold over-accumulation in both lines. Finally, additional alterations were found in the quinone group, with a series of positive (ubiquinone-9 and ubiquinone-10) and negative (α-tocopherol quinone and plastoquinone) changes, although they are statistically significant only in line 2B19.

The greatest alteration in fruit metabolite composition and abundance was revealed between breaker and Br+4, with enrichments mostly in phytoene, β-carotene and lycopene. At Br+4 (Figure 4C), carotenoids from phytoene to α- and β-carotene were consistently up-regulated (2- to 8-fold, approximately), with a similar trend to tocochromanols. Chlorophylls remained high, while all their catabolites were depleted. The consistency of the phenotype observed in the two edited *gf* lines was reinforced by a principal component analysis (PCA) of the isoprenoid data at Br+4 (Figure 4D) which showed the clear separation between the WT and *gf* genotypes, which were between themselves consistently similar. Changes in the volatile profile of *gf* fruits were less pronounced than those observed for isoprenoid compounds. The greatest alteration was again found at Br+4, which corresponds to the commercial ripening stage, where a PCA analysis showed a trend separating *gf* and WT fruits (Supplementary Figure 3 and Supplementary Data Files 3, 4), but few differences at the single compound level were significant and for many features a clear separation between edited and WT fruits was not identified. Specifically, line 12A41 showed an increase in apocarotenoid compounds (approximately 3-fold for compounds such as neral, geranial, β-cyclocitral, and β-damascenone), and both lines had increased levels of linalool oxide. In summary, the metabolic profiling of *gf* and WT ‘MoneyMaker’ plants pointed to a significant shift in isoprenoid metabolism and enrichment in valuable secondary compounds in *gf* fruits.

### The *gf* Phenotype Is Associated to Pathogen Resistance in Tomato

**FIGURE 5:**
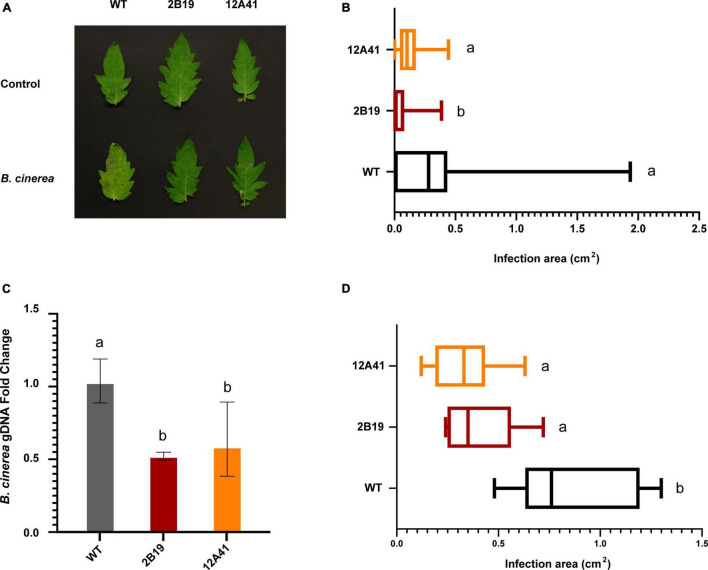
Association between *gf* mutations and pathogen resistance in tomato. **(A)** Infected and control tomato leaves 7 dpi. WT and *gf* tomato leaves infected with *B. cinerea* conidia or mock-infected with water (control). **(B)** Infection area of WT and *gf* tomato leaves infected with *B. cinerea*, 7 dpi. Bars indicate medians and extremes. Variables within the same statistical groups are marked with the same letters (Kruskal-Wallis ANOVA and Dunn’s *post-hoc* test, *p* < 0.05). **(C)** qPCR relative quantification of fungal gDNA from infected leaves. Values represent the fold change variation of *B. cinerea* genomic DNA in infected mutant *gf* leaves, relative to WT samples. Bars represent SD. Variables within the same statistical groups are marked with the same letters (ANOVA and Games-Howell *post-hoc* test, *p* < 0.001). **(D)** Infection area in full-ripe tomato fruits inoculated with *B. cinerea*, 7 dpi. Bars indicate medians and extremes. Variables within the same statistical groups are marked with the same letters (ANOVA and Games-Howell *post-hoc* test, *p* < 0.001).

Based on the evidence of an association between *gf*/*sgr* genotypes and pathogen resistance in Arabidopsis, *M. truncatula*, soybean and cucumber (Mecey et al., 2011; Ishiga et al., 2015; Chang et al., 2019; Wang et al., 2019), we tested the susceptibility of tomato leaves and fruits to *B. cinerea*, an important tomato pathogen causing the grey mold disease. A significant difference was observed for both organs. The extension of necrotic spots in tomato leaves was measured at 7 dpi (Figure 5A), finding a significant difference between WT and *gf* mutants of both lines, with infection area reduced on average by 83% in line 2B19 and by 66% in line 12A41 (Figure 5B). The proliferation of *B. cinerea* on infected tissues was estimated by qPCR amplification of the pathogen DNA (Figure 5C), finding a 50% reduction in *gf* leaves of both mutant lines. In ripe tomato fruits sampled at 7 dpi, the average infection area was reduced by 56% in line 2B19 and by 68% in line 12A41 (Figure 5D).

### Photosynthetic Activity of Edited *gf* Mutants

Parallel to the metabolite profiling of the 12A41 and 2B19 genotypes, analyses were conducted on two different T_2_ populations of the 12A line (12A26 and 12A50, both homozygous for a 4 bp deletion at the *GF* locus) to assess whether the CRISPR-induced *gf* mutations affected photosynthetic activity, net CO_2_ assimilation rate, PSII efficiency and chlorophyll content. Young and basal leaves were analyzed in WT and *gf* plants (Supplementary Figure 4). While chlorophyll content in basal leaves was approximately 15 times higher in *gf* than in WT leaves, net CO_2_ assimilation in young expanded leaves did not vary significantly between genotypes, and in basal leaves CO_2_ assimilation dropped in a similar manner in both *gf* and WT plants. The same trend was observed for PSII, where a slightly increased efficiency in young expanded leaves was not maintained with senescence. This is consistent with the “cosmetic” nature of *gf* mutations in terms of photosynthetic activity (Myers et al., 2018). The recapitulation of the *gf* phenotype also includes the unchanged photosynthetic capacity of mutant leaves, despite substantially increased chlorophyll accumulation.

## Discussion

### Specificity and Robustness of Cas9-Mediated Editing of *GF* in Tomato

The potential of CRISPR-based systems to introduce target mutations at selected loci in a specific and efficient manner is well established in tomato (Yu et al., 2017; Hashimoto et al., 2018; Li T. et al., 2018; Lu et al., 2021). In this work, we were able to assess the specificity, reproducibility and robustness for the editing of the *GF* locus. The consistency of the editing pattern is a valuable feature for breeding, since it makes this tool reliable for the introduction of this trait in different genetic backgrounds. In both analyzed cultivars, editing occurred with comparable efficiencies and resulted in the introduction of similar mutations, with a prevalence of small indels. In both ‘San Marzano’ and ‘MoneyMaker,’ the most common mutation was the introduction of a single nucleotide. We confirmed that no mutations were detectable at putative off-target loci. In plant research, off-target activity has been extensively investigated at different depths (from standard PCR to whole genome resequencing), highlighting how it is most often negligible, if not entirely undetectable (Hahn and Nekrasov, 2019). The very techniques used to obtain edited crops, such as *in vitro* regeneration, can in themselves induce a small number of random mutations often referred to as somaclonal variation. WGS of edited progenies detects a degree of variation consistent with such mutation rates (Nekrasov et al., 2017; Modrzejewski et al., 2020), and can be a valuable tool for the validation of an edited line with respect to its wild-type equivalent. An advantage of sexually propagated species, such as tomato, is that the extremely rare unwanted mutations can be easily segregated in selected progenies. Thanks to its precision and the possibility of segregating the targeted loci from the transgene, CRISPR represents the “cleanest” tool in our hands at this time to introduce tailored mutations in selected crops.

**FIGURE 6:**
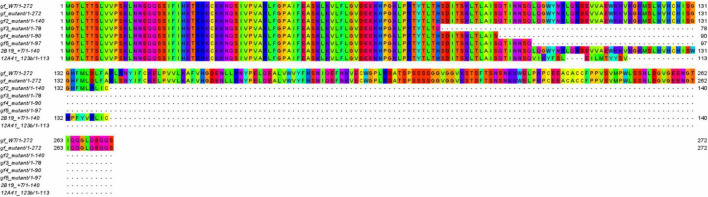
WT and mutated GF proteins. Alignment of the native and mutated GF proteins, including the *gf-gf^5^* spontaneous mutants and the CRISPR-generated mutants of lines 2B19 and 12A41. Alignments were obtained with T-Coffee Expresso and visualized with Jalview.

The robustness of a phenotype linked to a mutation in a gene depends on the consistency with which it can be introduced and maintained, and this is very dependent on the target locus. Gene editing techniques have helped establish the exact function of some genes whose mutations are linked to known phenotypes. An interesting example is that of important tomato ripening mutants such as *RIN*, *NOR*, and *CNR* (Gao et al., 2019, 2020): gene editing of these loci failed to fully recapitulate the phenotypes from which they were first described, and this in turn allowed a more accurate elucidation of their role and of the complex network which regulates fruit ripening. Edited lines can be considered truly isogenic lines and allow to reveal the phenotypes associated to a gene of interest by direct comparison with the line they derive from, and this contrasts with spontaneous mutants, for which original lines are often difficult to track, or with introgression lines that may contain other mutations linked to the one of interest. A targeted SDN-1 mutation allows to identify, in a more polished manner, the effects of a particular allele in a defined genetic background. We compared the predicted effects on the GF protein encoded by the edited *gf* alleles and those deriving from the five spontaneous mutations described by Barry and Pandey (2009; Figure 6). The mutation carried by line 2B19 (the insertion of a T at position 1750 in the *GF* locus) results in the introduction of a premature stop codon at positions 1781–1783; the *gf* ^2^ allele (the insertion of an A at position 1768) causes translation to stop at the same position. The 123 bp deletion of line 12A41 causes the elimination of a substantial region of exon 3, along with 23 nucleotides from the second intron. A similar rearrangement happens for the *gf*^5^ allele, where a 1,163 bp deletion removes the third and most of the fourth exons. In both cases, this results in the early termination of the protein. In *gf*^5^ mutants, GF is truncated at Q97; in 12A41 mutants the protein sequence diverges from the WT at the same Q97 position and ends at V113. 2B19 thus faithfully reproduces a mutation already present in the tomato gene pool, and 12A41 closely mimics another such mutation.

Our two lines, despite the structural differences of their mutations, show remarkable consistency in their phenotypes, something which is not true, for example, of *RIN* mutants obtained with CRISPR/Cas9, which showed different degrees of ripening delays. The reproducibility of a phenotype depends on various factors, including the role of the mutated protein product in cellular metabolism and the complex web of interactions in which it takes part, and locus architecture (for example, in *RIN* spontaneous mutants, *RIN* is truncated and fused in frame to a portion of the associated *MACROCALYX* locus, which causes it to switch from an activation to a repression function). For regulation, this means that the phenotypic consequences of a mutation should be carefully evaluated on a case-by-case basis, irrespective of the method used to obtain them. The ability to replicate a known mutation and, with it, a known phenotype, reinforces the argument of robustness for gene editing of the *GF* locus in tomato.

### Functional Aspects of *gf* Mutants for Fruit Quality and Resistance

The correlation between *gf* and increased carotenoid levels in tomato was described by Luo et al. (2013), Li X. et al. (2018), and Ma et al. (2022). In the model proposed by Luo et al. (2013), the phytoene synthase responsible for fruit carotenoid biosynthesis (PSY1) is negatively regulated by native GF, either post-transcriptionally by direct interaction, or indirectly through modulation of PIF1. Mutations in the *GF* locus lift this negative regulation, increasing phytoene synthase activity to levels comparable to those of a constitutively overexpressed PSY1: *gf* mutants accumulate higher levels of carotenoids, especially phytoene, lycopene and β-carotene. The metabolic profile of our edited fruits fits this model well, showing an increase of metabolites of the early steps of the carotenoid pathway. Comparable levels of lycopene and α- and β-carotene were also observed by Li X. et al. (2018) in *gf* tomato fruits at Br+7. Xanthophyll levels are less affected by the mutation: this could be explained by the fact that in *gf* plants the retention of chlorophyll does not correspond to increased or continued photosynthetic activity, making it unnecessary for cells to accumulate greater quantities of antenna pigments.

Chlorophyll retention is the most apparent metabolic change in our edited fruits. Chlorophyll *a* and *b* concentrations are greatly increased in mutants at all stages of ripening compared to the WT, but still decrease by the end of fruit ripening. Alternative mechanisms for chlorophyll degradation might be in place and, especially, two other loci in tomato might be implicated in starting chlorophyll breakdown. In addition to the described *GF* locus (Solyc08g080090), tomato encodes a staygreen-like (SGRL) protein (Solyc04g063240). SGRL proteins are known to intervene in chlorophyll degradation in leaves prior to senescence and under stress conditions (Rong et al., 2013; Sakuraba et al., 2014; Wu et al., 2016). Its specific role in tomato has not been elucidated yet, but SlSGRL is known to take part in chlorophyll breakdown in an ABA-dependent manner (Yang et al., 2020). Alignment of GF against the tomato proteome also identifies another locus (Solyc12g056480) which encodes a putative staygreen protein with a 72.69% similarity with GF (Matsuda et al., 2016). Unfortunately, it is not possible to make a direct comparison between our *gf* mutants and those of Luo et al. (2013) and Li X. et al. (2018) for chlorophyll retention. The first did not detect differences in chlorophyll accumulation between mutants and WT fruits, something which might be due to an incomplete disruption of GF activity by RNAi, while the second did not investigate this feature. However, the visual phenotype reported by Li X. et al. (2018) is indicative of chlorophyll retention during ripening.

**FIGURE 7:**
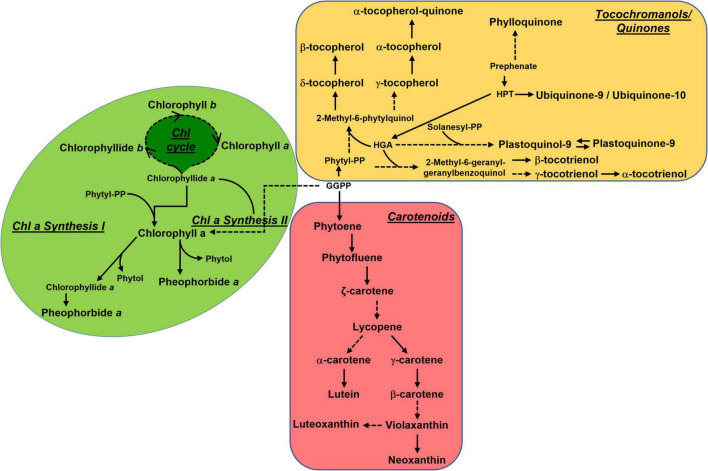
Overview of the main isoprenoid biosynthetic pathways. Metabolic relationships between the main isoprenoid classes (chlorophylls, carotenoids, and tocochromanols/quinones) referred to in this study. The metabolism of carotenoids, chlorophylls, and tocochromanols occurs in the plastid, while quinones are cytosolic.

Unexpectedly, we found that *gf* fruits accumulate considerably higher levels of vitamin E, especially at Br+4 and Br+8. High chlorophyll levels should in principle reduce vitamin E biosynthesis during fruit ripening, inhibiting the recycling of the chlorophyll-derived phytol toward tocochromanol biosynthesis. Previous studies have reported simultaneous increases in carotenoids and tocochromanols, as in the case of tomato fruits overexpressing PSY1 (Fraser et al., 2007), a biochemical phenotype occurring together with chlorophyll degradation. However, it is possible that the activation of the carotenoid pathway provides an abundance of precursors which are additionally channeled toward tocochromanol biosynthesis. An overview of the general isoprenoid pathways can be seen in Figure 7. Overall, these data suggest the existence of a complex and not yet fully understood equilibrium between chlorophyll synthesis/degradation, phytyl salvage pathway and synthesis and accumulation of other isoprenoid classes. However, it is worth noticing that these changes mainly affect plastidial (carotenoids and tocochromanols) rather than cytosolic (quinones) isoprenoids, thus indicating a reduced capacity of *gf* to affect carbon exchanges between MVA and MEP pathways. Notably, the acceptable daily intake (ADI) for vitamin E is 12 mg/die, and its concentration in tomato is on average 1 mg/100 g fw.^5^ The increase of vitamin E (especially α-tocopherol) in ripe fruits to up to around 10-fold represents a significant enrichment of this class of compounds with respect to WT fruits, meaning that just 120 g of *gf* fruits would be needed to meet the ADI.

The implication of *gf* in pathogen susceptibility in tomato had not been previously reported. Our findings in tomato fruits and leaves are consistent with the characterization of *staygreen*/*greenflesh* mutants in other crop species, namely soybean, *Medicago* and cucumber. A clear elucidation of the molecular mechanisms underlying this phenotype is still lacking. However, Wang et al. (2019) do propose a model in which *gf* is regarded as a *R* gene with a loss-of-susceptibility mechanism. In this model, the reduced chlorophyll breakdown of *gf* mutants limits the amount of ROS and phototoxic Chl catabolites produced upon pathogen infection, thus limiting symptom development. The fact that, despite not being involved in increased photosynthetic activity, light harvesting complexes are still present in plastids, together with an increase in carotenoids and vitamin E, might contribute to limiting oxidative damage and to the creation of a cellular environment capable of containing pathogen growth.

## Conclusion

In summary, our findings highlight how the *gf* mutation does not solely lead to a visible alteration of tomato fruit coloration, but also induces functional shifts which promote the accumulation of valuable health-promoting secondary compounds, which in turn also may have a positive impact on disease tolerance. While an increase in carotenoid levels had been already reported by other authors (Luo et al., 2013; Li X. et al., 2018), overaccumulation of vitamin E is a novel trait associated with these mutants, as is increased tolerance against fungal pathogens. The extensive functional characterization carried out on two isogenic lines which differ only based on the mutations induced at the *GF* locus allowed us to conclude that their phenotypes are consistent, with no allele-specific behavior. Also, the extensive genetic characterization carried out in two widely used tomato cultivars pointed to an extremely high editing efficiency for this particular locus, with conserved editing patterns between the two cultivars, and no unintended mutations at putative off-targets. All the data generated in this study makes gene editing of *GF* a highly reliable and valuable target for tomato breeding. We believe that careful, in-depth assessment of mutants is necessary for a rational evaluation of their functional characteristics as well as, in a regulatory perspective, of their impact. We hope that this operational strategy might serve as a roadmap for the integration of precise and reliable gene editing techniques to create valuable phenotypes in crop breeding.

## Data Availability Statement

The datasets presented in this study can be found in online repositories. The names of the repository/repositories and accession number(s) can be found below: NCBI BioProject, PRJNA615189.

## Author Contributions

SG, CC, AMo, GDi, DO, and AG designed the experiments. SG and AMo generated and grew the ‘MoneyMaker’ lines used in this study and performed genotyping experiments. AA and VG-C performed bioinformatic analyses. GDi and FS performed LC-MS analyses. JR performed GC-MS analyses. SG, GDi, FS, and JR analyzed the metabolomics data. GDo generated the ‘San Marzano’ mutants analyzed in this study. SG, EM-G, DV, and AMo performed Botrytis infection assays. MF-P, MC, and JG performed photosynthetic activity assays and analyzed the data. SG, DO, and AG drafted the manuscript. All authors read, revised, and approved the final manuscript.

## Conflict of Interest

The authors declare that the research was conducted in the absence of any commercial or financial relationships that could be construed as a potential conflict of interest.

## Publisher’s Note

All claims expressed in this article are solely those of the authors and do not necessarily represent those of their affiliated organizations, or those of the publisher, the editors and the reviewers. Any product that may be evaluated in this article, or claim that may be made by its manufacturer, is not guaranteed or endorsed by the publisher.

## Footnotes

1
https://eusage.eu/genome-search

2
https://benchling.com

3
http://www.rgenome.net/cas-offinder/

4
https://www.metaboanalyst.ca/

5
https://fdc.nal.usda.gov/
