# Supplementary material for: In-Depth Characterization of greenflesh Tomato Mutants Obtained by CRISPR/Cas9 Editing: A Case Study With Implications for Breeding and Regulation
Source: Front Plant Sci. 2022 Jul 11;13:936089. doi: 10.3389/fpls.2022.936089 (PMC9309892; doi:10.3389/fpls.2022.936089)
Supplement: Supplementary file 3 [file Table_3.DOCX]

**Supplementary Figure S1. Enrichment of edited alleles in heterozygous T_0_ plants.** Editing levels in 3 adult T_0_ plants, compared to the first screening carried out on rooting *ex vitro* plantlets. Editing efficiencies were calculated based on chromatogram decomposition using TIDE (<https://tide.nki.nl/>). Purple bars indicate editing levels in plantlets, and blue, green, orange and red bars indicate editing levels measured in two leaves and two fruits of adult plants, respectively. Editing percentage is reported for each sample/tissue below the graph.

**Supplementary Figure S2. Carotenoid composition across tomato fruit ripening.** Relative abundance of carotenoids in the fruits of WT MoneyMaker and 2B19 and 12A41 *gf* mutants from MG to eight days after breaker (Br+8).

**Supplementary Figure S3. Volatile profiles of WT and *gf* tomato fruits 4 days after breaker. A) Principal Component Analysis (PCA)** and **B) Hierarchical cluster analysis and heatmap representation** of the volatile metabolite composition of tomato fruits obtained by GC-MS. The heatmap represents the 30 most significantly different features, as determined by ANOVA (*p* < 0.05), while the PCA encompasses all features in the dataset. The data was obtained using Pearson’s distance measure and Ward’s minimum variance method. Red indicates up-regulated and blue indicates down-regulated features.

**Supplementary Figure S4. Photosynthetic activity in WT and *gf* leaves.** Variation among lines in **a) net CO_2_ assimilation rate (A_N_)**, **b) efficiency of photosystem II (FPSII)** and **c) SPAD values**. Data are means ± S.E. (*n* = 4). Filled bars refer to young fully expanded leaves located in a high position in the plant canopy and unfilled bars to basal leaves. Asterisks in unfilled bars denote differences between leaves levels for the same genotype, capital letters denote significant differences among genotypes for leaves located in a high position in the plant canopy and lowercase letters denote differences among genotypes for basal leaves by one-way ANOVA after Duncan post hoc test (*p*-value < 0.05).
