# Supplementary material for: In-Depth Characterization of greenflesh Tomato Mutants Obtained by CRISPR/Cas9 Editing: A Case Study With Implications for Breeding and Regulation
Source: Front Plant Sci. 2022 Jul 11;13:936089. doi: 10.3389/fpls.2022.936089 (PMC9309892; doi:10.3389/fpls.2022.936089)
Supplement: Supplementary file 4 [file Table_4.DOCX]

**Supplementary Table S1:** GB parts used and generated in this work.

| **GB parts** | | | |
| --- | --- | --- | --- |
| **GB database ID** | **Name** | **Level** | **Category** |
| GB1001 | pUPD U6-26 | 0 | Promoter |
| GB0645 | pUPD sgRNA | 0 | Other |
| GB4586 | α1 PU6-26:gRNA:scaffold | 1 | Intermediary construct |
| GB0639 | α2 P35S:hCas9:Tnos | 1 | TU |
| GB4587 | Ω2 PU6-26:gRNA:scaffold - P35S:hCas9:Tnos | >1 | Intermediary construct |
| GB1181 | Ω1R Tnos:nptII:Pnos | 1 | TU |
| GB4588 | α2 Tnos:nptII:Pnos - PU6-26:gRNA:scaffold - P35S:hCas9:Tnos | >1 | MODULE |

**Supplementary Table S2:** Putative off-target sites for the selected *gf* gRNA.

| **Off-target** | **Chromosome** | **Position** | **Sequence** | **Annotation** |
| --- | --- | --- | --- | --- |
| 1 | 6 | 7491701 | agtCATTGCgAtATTAGTGGGGG | non coding |
| 2 | 2 | 30647910 | GcaaATTGCtcCATTAGTGGTGG | non coding |
| 3 | 2 | 34542804 | GTtggTgGaCACATTAGTGGTGG | cannabidiolic acid synthase (accession XM_004233099) |
| 4 | 6 | 38237927 | GgCCgTTGCCACATATAcTGGTGG | probable plastidic glucose transporter 3  (accession XM_010324389) |
| 5 | 1 | 40900162 | tTCCAcTtCC-CATTAGTGGTGG | non coding |

**Supplementary Table S3:** oligonucleotides and primers.

| **gRNA oligonucleotides** (GoldenBraid adapters in bold) | |
| --- | --- |
| ***gf* gRNA strand +** | **ATT**GTCCATTGCCACATTAGTGG |
| ***gf* gRNA strand -** | **AAAC**CCACTAATGTGGCAATGGA |
| **Sanger genotyping primers** | |
| ***gf* forward** | GGGTCTGGGCCAAAACTACT |
| ***gf* reverse** | ACAGGCACAAGCCTCTTCAC |
| ***hCas9* forward** | AGGTGGCGTACCATGAAAAG |
| ***hCas9* reverse** | TGTTTGCGCAACAGATCTTC |
| **Illumina Amplicon Sequencing genotyping** (Illumina adapters in bold) | |
| ***gf* forward** | **TCGTCGGCAGCGTCAGATGTGTATAAGAGACAG**ATCTTGAATCTGCATCTACATCG |
| ***gf* reverse** | **GTCTCGTGGGCTCGGAGATGTGTATAAGAGACAG**CACAGGGAGTTCTTTGCAGA |
| **OT1 forward** | **TCGTCGGCAGCGTCAGATGTGTATAAGAGACAG**GATGTGAAGATTGTAGTTTG |
| **OT1 reverse** | **GTCTCGTGGGCTCGGAGATGTGTATAAGAGACAG**GCACAGTAAATAGGGTGAAG |
| **OT2 forward** | **TCGTCGGCAGCGTCAGATGTGTATAAGAGACAG**CAAAAGGGCATTCGAACT |
| **OT2 reverse** | **GTCTCGTGGGCTCGGAGATGTGTATAAGAGACAG**TATACAACGCCAGAGTTTCAG |
| **OT3 forward** | **TCGTCGGCAGCGTCAGATGTGTATAAGAGACAG**TTCTGACAACCCCTTTTTC |
| **OT3 reverse** | **GTCTCGTGGGCTCGGAGATGTGTATAAGAGACAG**TGTTATCAGCAGCAATACCA |
| **OT4 forward** | **TCGTCGGCAGCGTCAGATGTGTATAAGAGACAG**GTATAATACTCTTCTAGTGTACCA |
| **OT4 reverse** | **GTCTCGTGGGCTCGGAGATGTGTATAAGAGACAG**GTCATACCCAAGATGGTA |
| **OT5 forward** | **TCGTCGGCAGCGTCAGATGTGTATAAGAGACAG**CTCCGCCTTTTTTCATCAAATCC |
| **OT5 reverse** | **GTCTCGTGGGCTCGGAGATGTGTATAAGAGACAG**TTTGGTTGGCGAATGGCA |

**Supplementary Table S4:** genotyping of 2B19 and 12A41 T_1_ individuals and of two individuals from their T_2_ progenies. OT frequencies are expressed as % of mutated reads from Illumina Amplicon Sequencing. ‘-’ indicates non-detectable PCR products.

| **Line** | **Cas9** | ***gf*** | **OT1** | **OT2** | **OT3** | **OT4** | **OT5** |
| --- | --- | --- | --- | --- | --- | --- | --- |
| 2B19 | - | +T/+T | - | 0 | - | 0.7 | 0.49 |
| 2B19_n | - | +T/+T | 0 | 0.08 | 0 | 0.13 | 0.64 |
| 12A41 | - | -123/-123 | 0 | 0 | 1.28 | 0.8 | 0.62 |
| 12A41_n | - | -123/-123 | 0 | 0.11 | 0.34 | 0.47 | 1.02 |
