# Supplementary material for: In-Depth Characterization of greenflesh Tomato Mutants Obtained by CRISPR/Cas9 Editing: A Case Study With Implications for Breeding and Regulation
Source: Front Plant Sci. 2022 Jul 11;13:936089. doi: 10.3389/fpls.2022.936089 (PMC9309892; doi:10.3389/fpls.2022.936089)
Supplement: Supplementary file 5 [file Presentation_1.PPTX]

## Slide 1
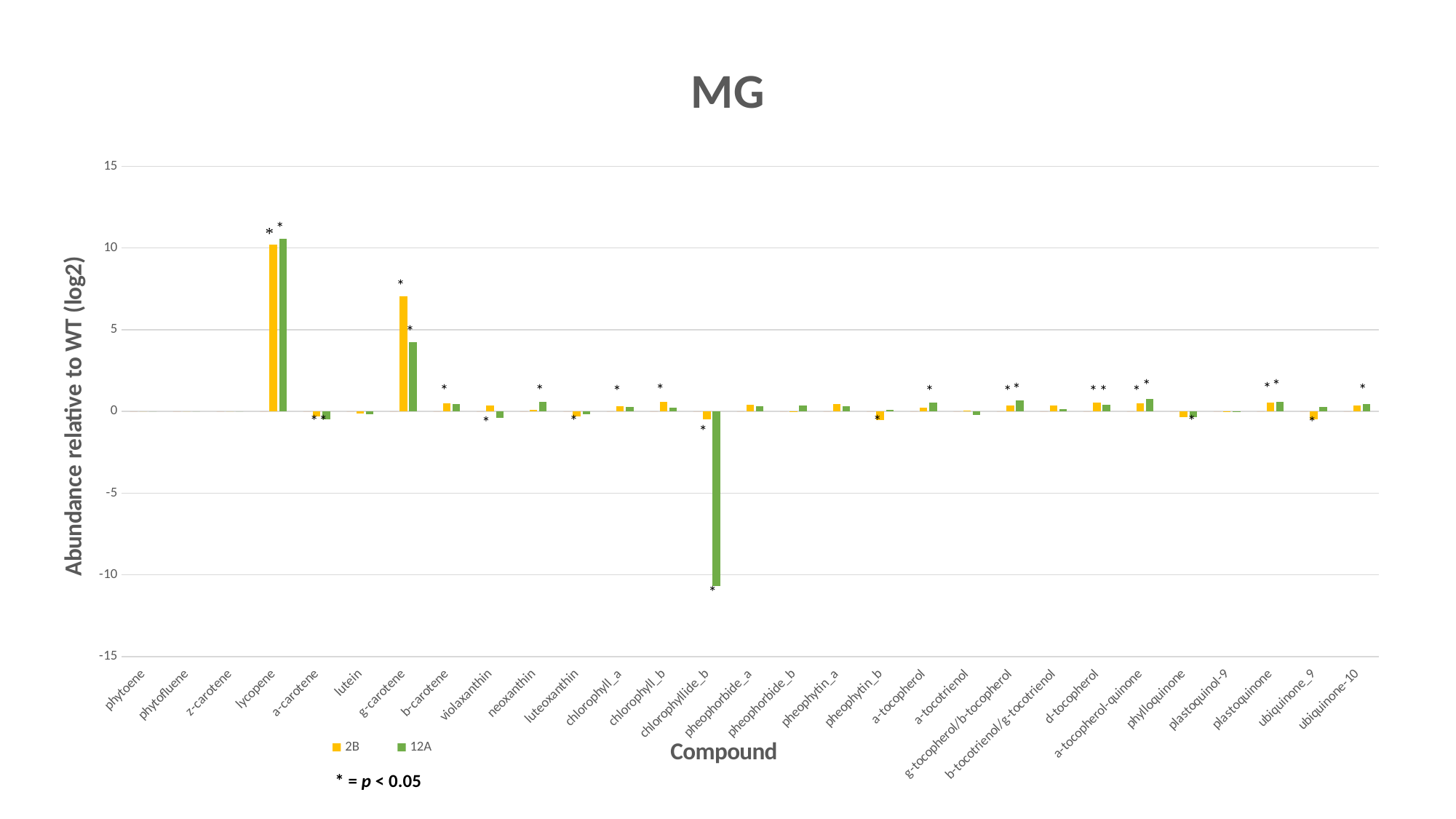

### Chart: MG
| Category | WT | 2B | 12A |
|---|---|---|---|
| phytoene | 0.0 | 0.0 | 0.0 |
| phytofluene | 0.0 | 0.0 | 0.0 |
| z-carotene | 0.0 | 0.0 | 0.0 |
| lycopene | 0.0 | 10.225891767934836 | 10.546789410494647 |
| a-carotene | 0.0 | -0.30771356720803705 | -0.463179691574729 |
| lutein | 0.0 | -0.14585646961825385 | -0.1839762514081608 |
| g-carotene | 0.0 | 7.04111567183565 | 4.214269376745152 |
| b-carotene | 0.0 | 0.512575949140432 | 0.4605720549741219 |
| violaxanthin | 0.0 | 0.3425181919897441 | -0.3846066147498525 |
| neoxanthin | 0.0 | 0.11869177800268697 | 0.5720232686714294 |
| luteoxanthin | 0.0 | -0.3250258196782737 | -0.16847413107914574 |
| chlorophyll_a | 0.0 | 0.33718436367553656 | 0.2555529057634152 |
| chlorophyll_b | 0.0 | 0.6047706728482669 | 0.24182993935768612 |
| chlorophyllide_b | 0.0 | -0.4968063566994279 | -10.670113193801996 |
| pheophorbide_a | 0.0 | 0.3888996688201694 | 0.3266554579834063 |
| pheophorbide_b | 0.0 | -0.02473886566294874 | 0.35949998894064156 |
| pheophytin_a | 0.0 | 0.46680078195510066 | 0.331726769192271 |
| pheophytin_b | 0.0 | -0.5376347384088372 | 0.10879497644981731 |
| a-tocopherol | 0.0 | 0.22845056466341168 | 0.5228890345019331 |
| a-tocotrienol | 0.0 | 0.049978448462481555 | -0.23301327256187837 |
| g-tocopherol/b-tocopherol | 0.0 | 0.37712747961156073 | 0.6728584337869328 |
| b-tocotrienol/g-tocotrienol | 0.0 | 0.37498823074814497 | 0.1568641663659733 |
| d-tocopherol | 0.0 | 0.5495166790687626 | 0.3989215747048433 |
| a-tocopherol-quinone | 0.0 | 0.5165113991601729 | 0.7446393792279585 |
| phylloquinone | 0.0 | -0.33637120071194165 | -0.35182522219961193 |
| plastoquinol-9 | 0.0 | 0.022480768270388123 | 0.026751798257071958 |
| plastoquinone | 0.0 | 0.5209335388157507 | 0.5693024156552094 |
| ubiquinone_9 | 0.0 | -0.4656234453651354 | 0.28830470859196194 |
| ubiquinone-10 | 0.0 | 0.3414010939435952 | 0.4660110182105992 |
* = p < 0.05

## Slide 2
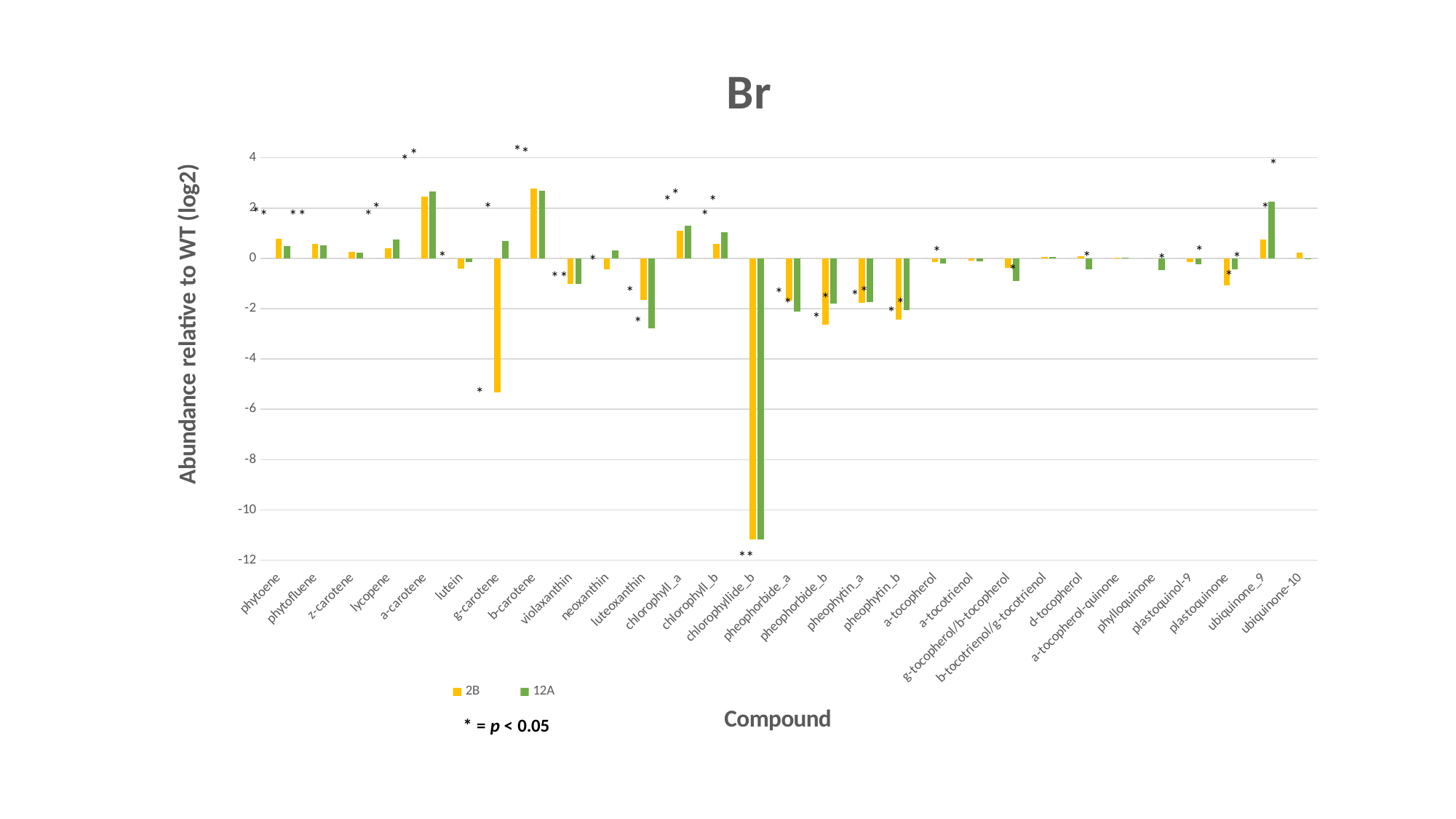

### Chart: Br
| Category | WT | 2B | 12A |
|---|---|---|---|
| phytoene | 0.0 | 0.7712430313783692 | 0.4855288695836286 |
| phytofluene | 0.0 | 0.5864073530885651 | 0.5084576896777154 |
| z-carotene | 0.0 | 0.2624195917050086 | 0.23053976957826658 |
| lycopene | 0.0 | 0.4070881276095788 | 0.7573892802369844 |
| a-carotene | 0.0 | 2.4585334236309473 | 2.6469716174080333 |
| lutein | 0.0 | -0.40767395128056605 | -0.14968743806028106 |
| g-carotene | 0.0 | -5.331517657607153 | 0.6826703220703905 |
| b-carotene | 0.0 | 2.7739202225404136 | 2.7008183653009903 |
| violaxanthin | 0.0 | -1.0230363310997694 | -1.029593365107955 |
| neoxanthin | 0.0 | -0.4246980366422225 | 0.31361628023725835 |
| luteoxanthin | 0.0 | -1.6584088293590933 | -2.7955945021724955 |
| chlorophyll_a | 0.0 | 1.091928906859639 | 1.3005060644193287 |
| chlorophyll_b | 0.0 | 0.5864856209679454 | 1.0493576471707735 |
| chlorophyllide_b | 0.0 | -11.162489320448534 | -11.162489320448534 |
| pheophorbide_a | 0.0 | -1.6813067048007193 | -2.114022281060768 |
| pheophorbide_b | 0.0 | -2.6388863092143944 | -1.7971959081017046 |
| pheophytin_a | 0.0 | -1.7733038189082742 | -1.7256664466608194 |
| pheophytin_b | 0.0 | -2.423437171822867 | -2.066015780463822 |
| a-tocopherol | 0.0 | -0.13983357657801557 | -0.2099425030939729 |
| a-tocotrienol | 0.0 | -0.077119282564209 | -0.11428893210224861 |
| g-tocopherol/b-tocopherol | 0.0 | -0.37924093374646334 | -0.8901118020552536 |
| b-tocotrienol/g-tocotrienol | 0.0 | 0.04179037087440559 | 0.053736441015250956 |
| d-tocopherol | 0.0 | 0.07515823474445672 | -0.4253166665523388 |
| a-tocopherol-quinone | 0.0 | 0.022195884704473 | 0.014566925325147232 |
| phylloquinone | 0.0 | 0.0010324897261696515 | -0.4633236555813679 |
| plastoquinol-9 | 0.0 | -0.1616855802290841 | -0.22283491262473648 |
| plastoquinone | 0.0 | -1.0697928504969476 | -0.43471478297427785 |
| ubiquinone_9 | 0.0 | 0.7427749528720304 | 2.2447437396071703 |
| ubiquinone-10 | 0.0 | 0.22692147714275077 | -0.011919626284656832 |*

## Slide 3
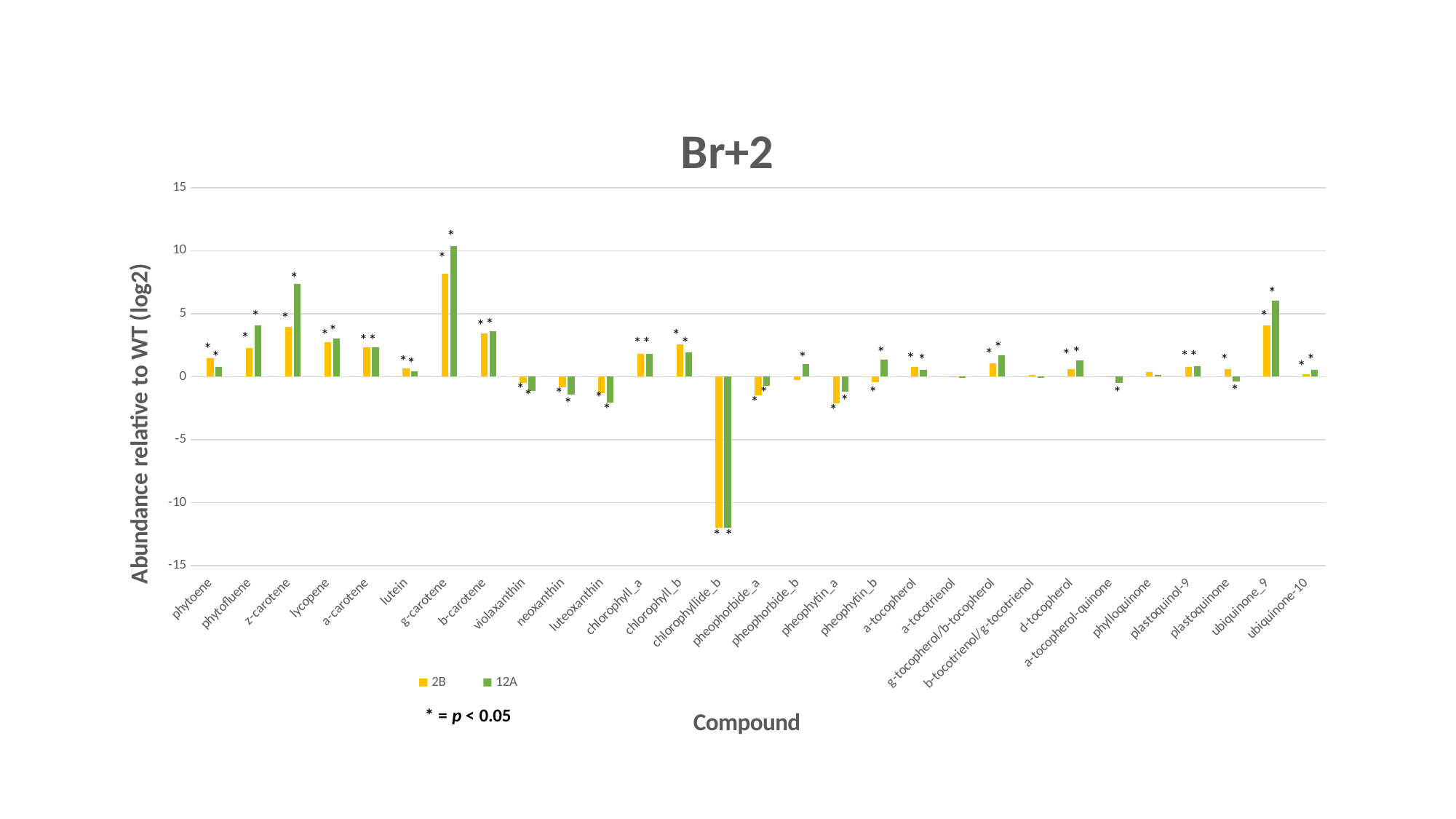

### Chart: Br+2
| Category | WT | 2B | 12A |
|---|---|---|---|
| phytoene | 0.0 | 1.4814184043430667 | 0.8008761421379453 |
| phytofluene | 0.0 | 2.2516852555218687 | 4.088949835747905 |
| z-carotene | 0.0 | 3.949047951213477 | 7.3313565520297015 |
| lycopene | 0.0 | 2.7446608274244664 | 3.0386822685520647 |
| a-carotene | 0.0 | 2.348508310011298 | 2.3165439859832913 |
| lutein | 0.0 | 0.648660627902514 | 0.41545270099585824 |
| g-carotene | 0.0 | 8.188788473722127 | 10.371839397286355 |
| b-carotene | 0.0 | 3.4324010396840343 | 3.6210096890881163 |
| violaxanthin | 0.0 | -0.5000313546445992 | -1.1245789710939222 |
| neoxanthin | 0.0 | -0.8586449207593847 | -1.445814276006179 |
| luteoxanthin | 0.0 | -1.2987384091800043 | -2.0397942458146034 |
| chlorophyll_a | 0.0 | 1.7934545145062906 | 1.8259728778478073 |
| chlorophyll_b | 0.0 | 2.5356440931019906 | 1.9198349055315778 |
| chlorophyllide_b | 0.0 | -12.01786487173173 | -12.01786487173173 |
| pheophorbide_a | 0.0 | -1.4774389505381271 | -0.7557301056547625 |
| pheophorbide_b | 0.0 | -0.2716313714960858 | 0.9946760205292917 |
| pheophytin_a | 0.0 | -2.094859100859277 | -1.1852694923560094 |
| pheophytin_b | 0.0 | -0.4171606617036437 | 1.3292937045572406 |
| a-tocopherol | 0.0 | 0.7541929351942607 | 0.5573112793401077 |
| a-tocotrienol | 0.0 | 0.0291845390358317 | -0.07807684665569309 |
| g-tocopherol/b-tocopherol | 0.0 | 1.081320364287753 | 1.704613153302093 |
| b-tocotrienol/g-tocotrienol | 0.0 | 0.12785580399470445 | -0.07822825184372284 |
| d-tocopherol | 0.0 | 0.6132978249755979 | 1.287141965717975 |
| a-tocopherol-quinone | 0.0 | -0.005093778257963649 | -0.4964042744543118 |
| phylloquinone | 0.0 | 0.38219552443849986 | 0.12576527015062622 |
| plastoquinol-9 | 0.0 | 0.7874864884016551 | 0.8287231776995573 |
| plastoquinone | 0.0 | 0.6227133587119168 | -0.35604264725047047 |
| ubiquinone_9 | 0.0 | 4.047874588242335 | 6.021919784977612 |
| ubiquinone-10 | 0.0 | 0.203932724841058 | 0.5531597420976306 |

## Slide 4
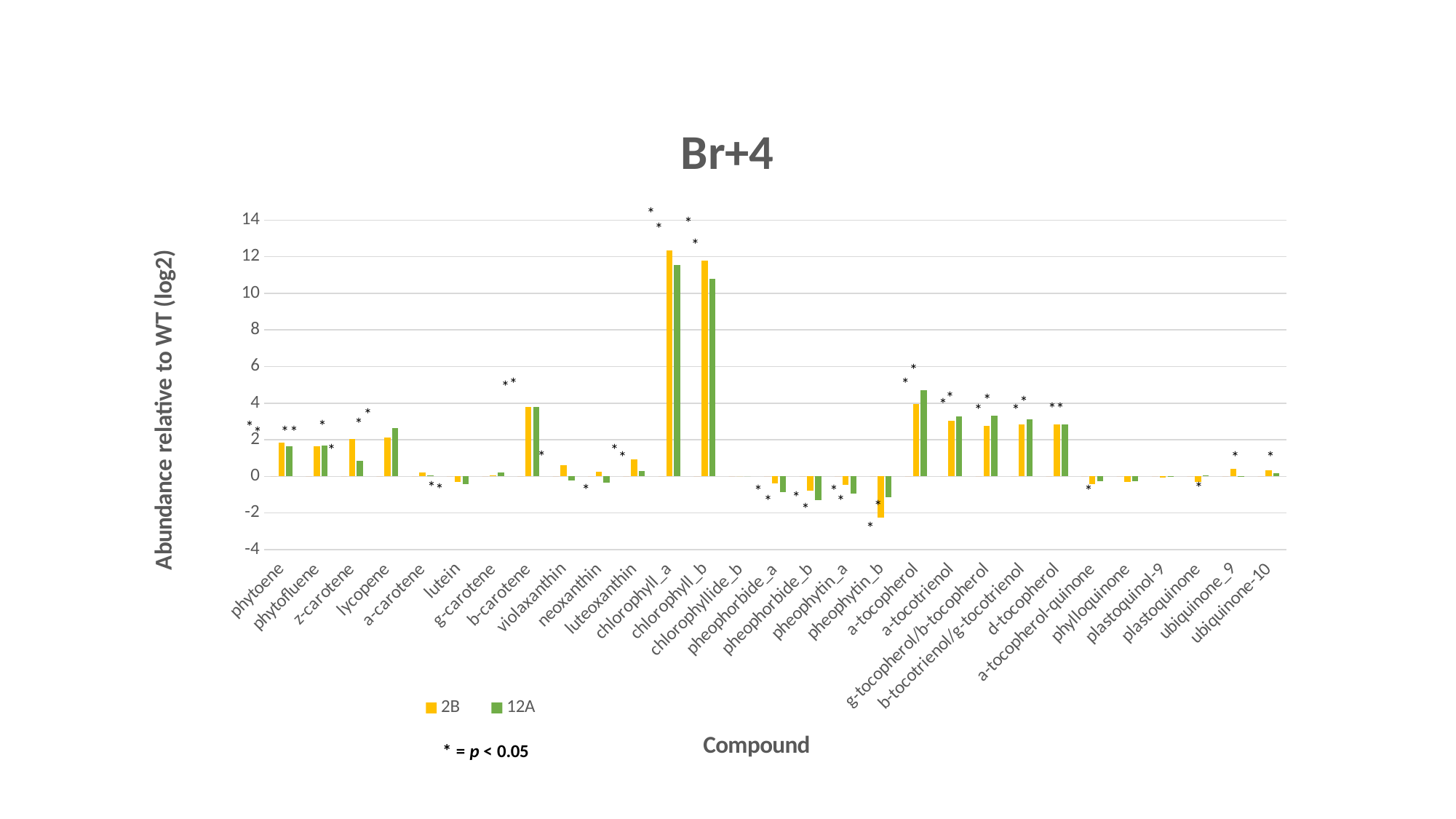

### Chart: Br+4
| Category | WT | 2B | 12A |
|---|---|---|---|
| phytoene | 0.0 | 1.839199619053629 | 1.624846990200734 |
| phytofluene | 0.0 | 1.6564952004180884 | 1.6650243839100354 |
| z-carotene | 0.0 | 2.047550589597919 | 0.8607936570640973 |
| lycopene | 0.0 | 2.131421893825608 | 2.6497705016845887 |
| a-carotene | 0.0 | 0.20957690933335638 | 0.05998167944222107 |
| lutein | 0.0 | -0.3010923127238971 | -0.4105770088355564 |
| g-carotene | 0.0 | 0.033340207689360496 | 0.20913290780934257 |
| b-carotene | 0.0 | 3.7748754859526503 | 3.798319449243472 |
| violaxanthin | 0.0 | 0.616851861769181 | -0.2479498407900066 |
| neoxanthin | 0.0 | 0.23394328170779316 | -0.34445995616230796 |
| luteoxanthin | 0.0 | 0.9255851670144664 | 0.2914429860669951 |
| chlorophyll_a | 0.0 | 12.354671005761096 | 11.534186843020485 |
| chlorophyll_b | 0.0 | 11.805346892036866 | 10.791952231657575 |
| chlorophyllide_b | 0.0 | 0.0 | 0.0 |
| pheophorbide_a | 0.0 | -0.37867134068145963 | -0.867173577251475 |
| pheophorbide_b | 0.0 | -0.7833673338747369 | -1.2849890286411172 |
| pheophytin_a | 0.0 | -0.4515223208058988 | -0.9507834274179675 |
| pheophytin_b | 0.0 | -2.271598356710424 | -1.134984220830013 |
| a-tocopherol | 0.0 | 3.950830760307193 | 4.701271337111749 |
| a-tocotrienol | 0.0 | 3.0496304446789733 | 3.272527161614415 |
| g-tocopherol/b-tocopherol | 0.0 | 2.750168222934019 | 3.3084061394232696 |
| b-tocotrienol/g-tocotrienol | 0.0 | 2.8233326418326463 | 3.1201584875105843 |
| d-tocopherol | 0.0 | 2.8526086567462983 | 2.844815350133139 |
| a-tocopherol-quinone | 0.0 | -0.4407630585390119 | -0.2633384736079822 |
| phylloquinone | 0.0 | -0.2941417040426239 | -0.2576573812467295 |
| plastoquinol-9 | 0.0 | -0.05302747007603015 | -0.04610470680575823 |
| plastoquinone | 0.0 | -0.3188624577183394 | 0.07007529576668581 |
| ubiquinone_9 | 0.0 | 0.41305318432064353 | -0.024809816717168077 |
| ubiquinone-10 | 0.0 | 0.3158360283640379 | 0.17565819271752697 |

## Slide 5
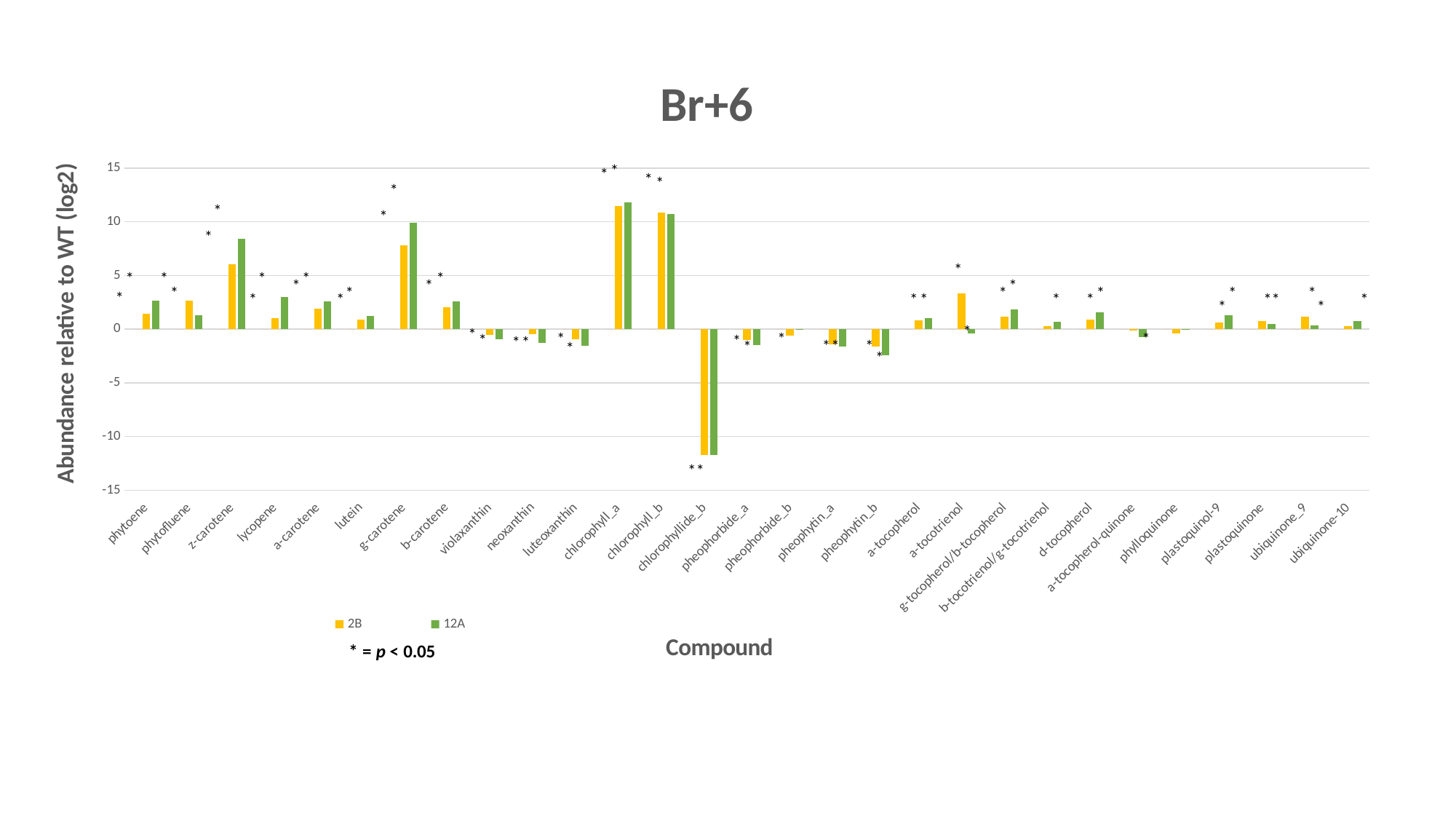

### Chart: Br+6
| Category | WT | 2B | 12A |
|---|---|---|---|
| phytoene | 0.0 | 1.4112661240616007 | 2.650409486516449 |
| phytofluene | 0.0 | 2.685206496468009 | 1.3085490292633009 |
| z-carotene | 0.0 | 6.032841754407234 | 8.434060734882467 |
| lycopene | 0.0 | 1.0516540790978743 | 3.0247218765094206 |
| a-carotene | 0.0 | 1.8766792653699398 | 2.6220858105980094 |
| lutein | 0.0 | 0.9031962562796952 | 1.2408235933532241 |
| g-carotene | 0.0 | 7.8182825280622446 | 9.9320345929439 |
| b-carotene | 0.0 | 2.0486716311741815 | 2.5589032920741976 |
| violaxanthin | 0.0 | -0.5020518412549233 | -0.9103230127659208 |
| neoxanthin | 0.0 | -0.4708123794936976 | -1.245328295220819 |
| luteoxanthin | 0.0 | -0.9566400531867401 | -1.5402105330153932 |
| chlorophyll_a | 0.0 | 11.4914935874491 | 11.814674931981376 |
| chlorophyll_b | 0.0 | 10.872476862790394 | 10.700220530195224 |
| chlorophyllide_b | 0.0 | -11.73820186542834 | -11.73820186542834 |
| pheophorbide_a | 0.0 | -1.0318970736458675 | -1.4698144478826956 |
| pheophorbide_b | 0.0 | -0.5791010343952526 | -0.08983485695918875 |
| pheophytin_a | 0.0 | -1.40415796346857 | -1.6205020617972719 |
| pheophytin_b | 0.0 | -1.605534691398315 | -2.448772694423289 |
| a-tocopherol | 0.0 | 0.8498311407282382 | 1.0144002615611791 |
| a-tocotrienol | 0.0 | 3.3162307014123296 | -0.4146248796988026 |
| g-tocopherol/b-tocopherol | 0.0 | 1.1536200080256371 | 1.8757166249470771 |
| b-tocotrienol/g-tocotrienol | 0.0 | 0.2755695244113927 | 0.6896824919310829 |
| d-tocopherol | 0.0 | 0.8750375104938325 | 1.5938185103189726 |
| a-tocopherol-quinone | 0.0 | -0.14098679281429943 | -0.7231652991498149 |
| phylloquinone | 0.0 | -0.3980207872322066 | -0.006853727916379213 |
| plastoquinol-9 | 0.0 | 0.6223419078229486 | 1.2789991818402555 |
| plastoquinone | 0.0 | 0.7672348208334353 | 0.4678140832575017 |
| ubiquinone_9 | 0.0 | 1.173940910737895 | 0.3828741620424145 |
| ubiquinone-10 | 0.0 | 0.286149438226995 | 0.7875643765063038 |*

## Slide 6
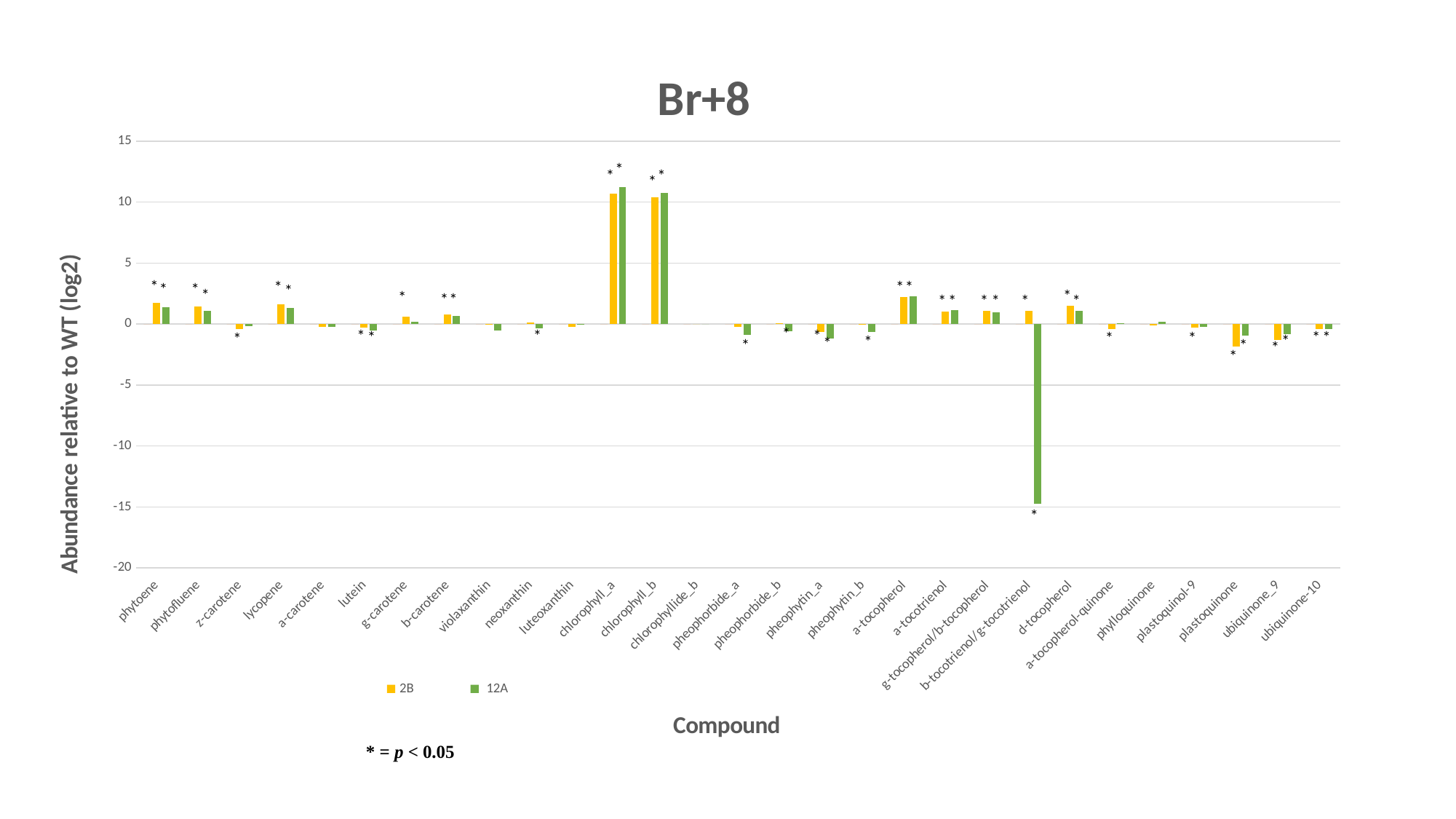

### Chart: Br+8
| Category | WT | 2B | 12A |
|---|---|---|---|
| phytoene | 0.0 | 1.7195170641408062 | 1.412170344814472 |
| phytofluene | 0.0 | 1.4525064035123332 | 1.0989339221020515 |
| z-carotene | 0.0 | -0.4009780665103195 | -0.14958353630545165 |
| lycopene | 0.0 | 1.6409915700125286 | 1.3391973085536872 |
| a-carotene | 0.0 | -0.2309102675573261 | -0.20364487978463802 |
| lutein | 0.0 | -0.2632641250697251 | -0.5037857374999154 |
| g-carotene | 0.0 | 0.6326142399265047 | 0.17747812228578325 |
| b-carotene | 0.0 | 0.771656077559961 | 0.6915213341901238 |
| violaxanthin | 0.0 | -0.07625795722403268 | -0.5540415404929792 |
| neoxanthin | 0.0 | 0.10856987675332014 | -0.3563523550639974 |
| luteoxanthin | 0.0 | -0.22693205082675627 | 0.014034598623239218 |
| chlorophyll_a | 0.0 | 10.696955386668899 | 11.22803864697155 |
| chlorophyll_b | 0.0 | 10.408584145015652 | 10.73003181638429 |
| chlorophyllide_b | 0.0 | 0.0 | 0.0 |
| pheophorbide_a | 0.0 | -0.254371010827199 | -0.9033188389917814 |
| pheophorbide_b | 0.0 | 0.07812146793453377 | -0.5860822116281752 |
| pheophytin_a | 0.0 | -0.6199758817026286 | -1.2137127608489726 |
| pheophytin_b | 0.0 | -0.07882092840350009 | -0.6779033283240964 |
| a-tocopherol | 0.0 | 2.246204349924806 | 2.2537114424271496 |
| a-tocotrienol | 0.0 | 1.0199852352992138 | 1.1184918042874374 |
| g-tocopherol/b-tocopherol | 0.0 | 1.070198839089169 | 0.9945894226944128 |
| b-tocotrienol/g-tocotrienol | 0.0 | 1.0859391161431255 | -14.767391085212541 |
| d-tocopherol | 0.0 | 1.4945054000596192 | 1.0890910014381063 |
| a-tocopherol-quinone | 0.0 | -0.39465322783210266 | 0.09376688681815157 |
| phylloquinone | 0.0 | -0.12849509820871247 | 0.20086122759976824 |
| plastoquinol-9 | 0.0 | -0.30869903600548204 | -0.25224594298405584 |
| plastoquinone | 0.0 | -1.8362134860435677 | -0.945855612728194 |
| ubiquinone_9 | 0.0 | -1.3000521884558436 | -0.8510201597010209 |
| ubiquinone-10 | 0.0 | -0.4148535259948471 | -0.3808013551845778 |
