# Supplementary figures and images for: In-Depth Characterization of greenflesh Tomato Mutants Obtained by CRISPR/Cas9 Editing: A Case Study With Implications for Breeding and Regulation
Source: Front Plant Sci. 2022 Jul 11;13:936089. doi: 10.3389/fpls.2022.936089 (PMC9309892; doi:10.3389/fpls.2022.936089)

## Slide 1
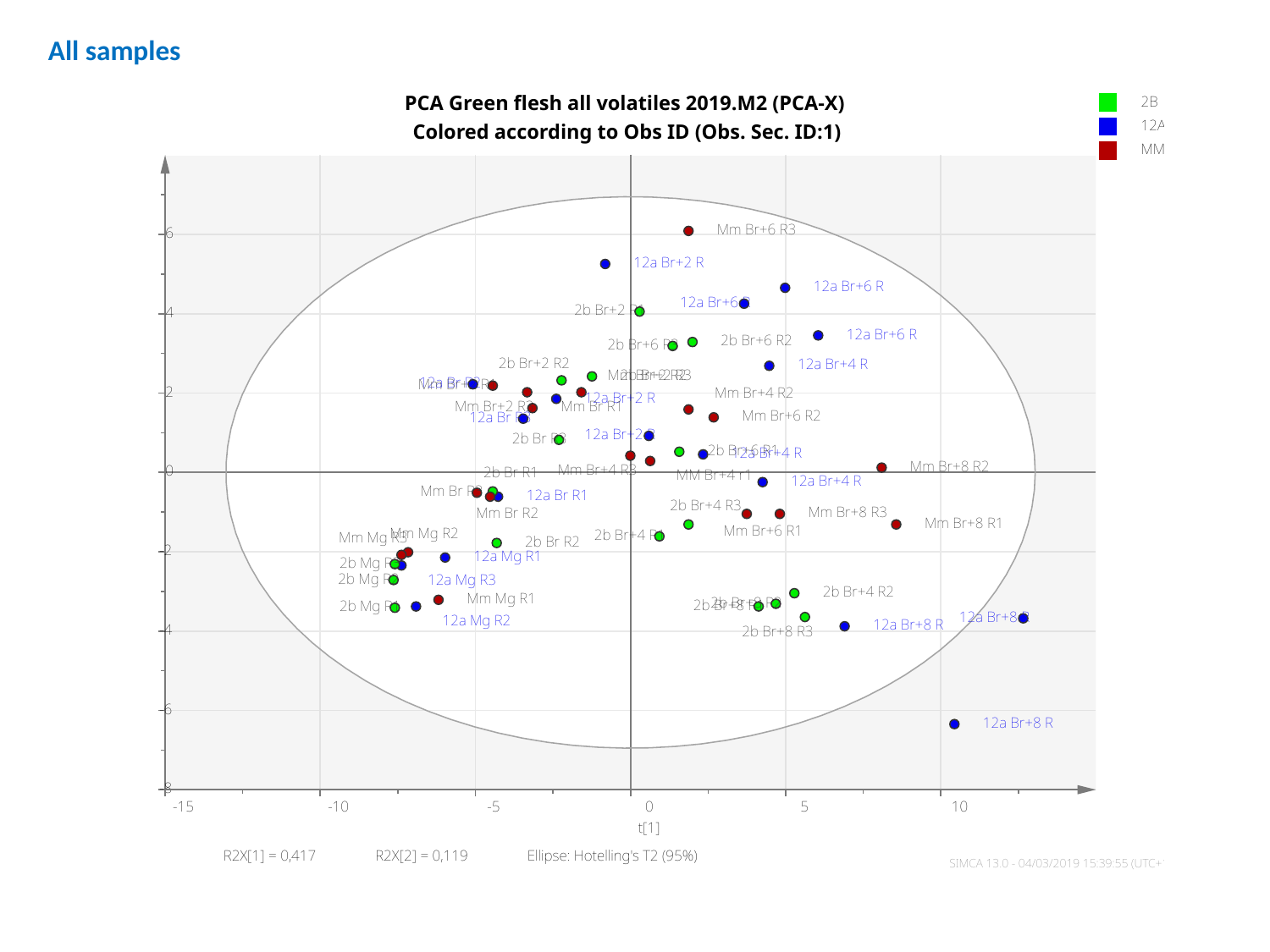

All samples

## Slide 2
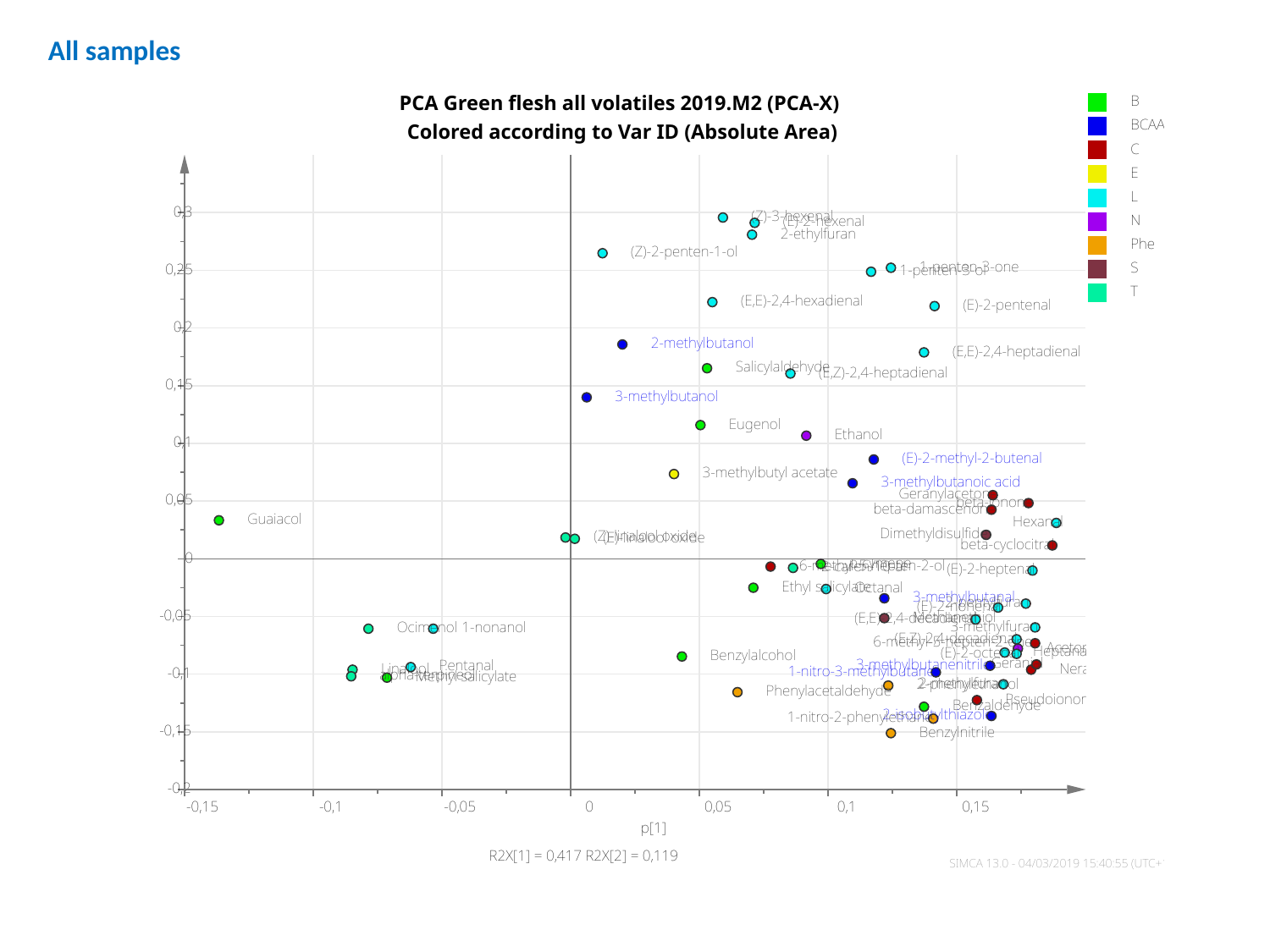

All samples

## Slide 3
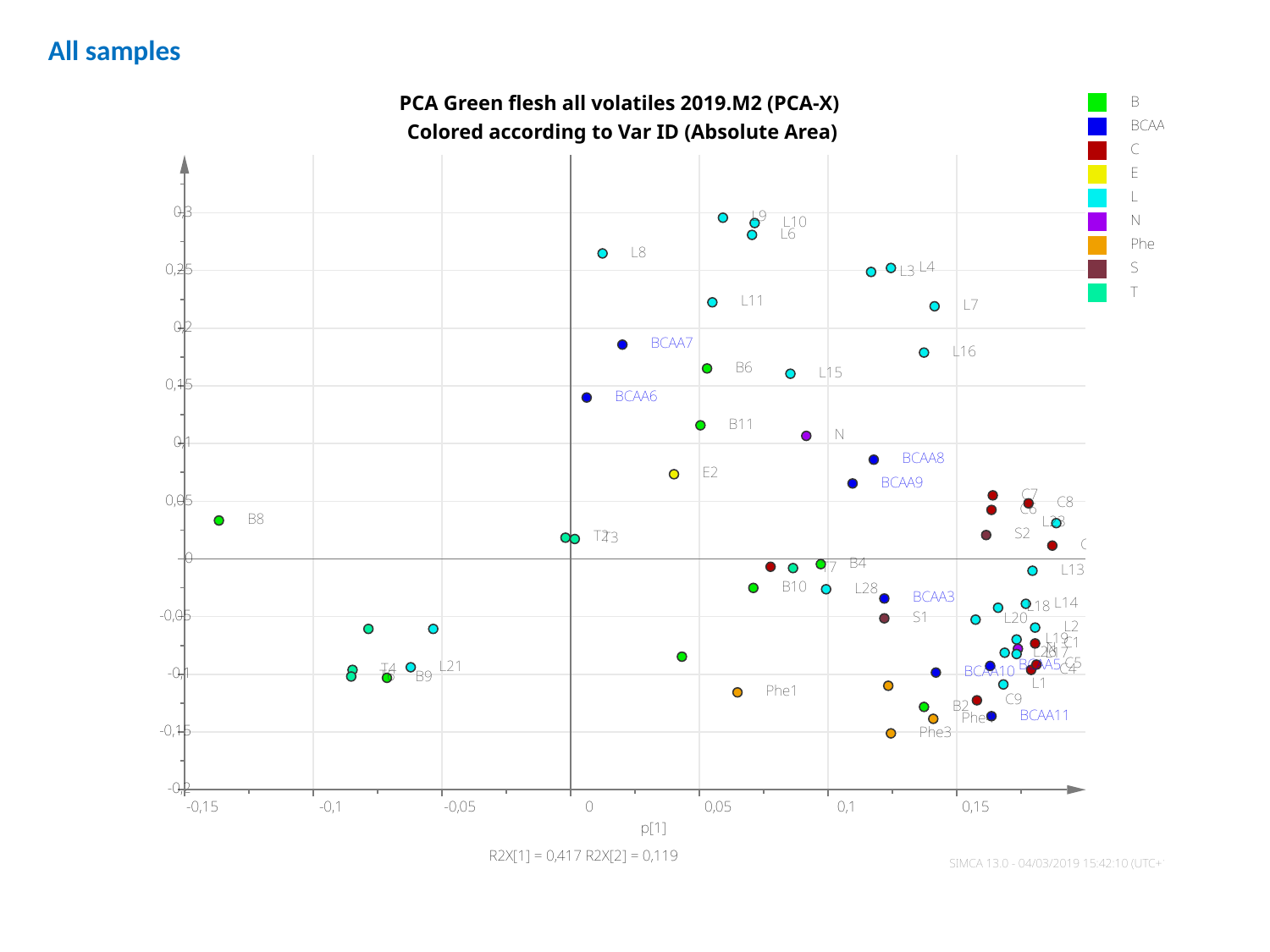

All samples

## Slide 4
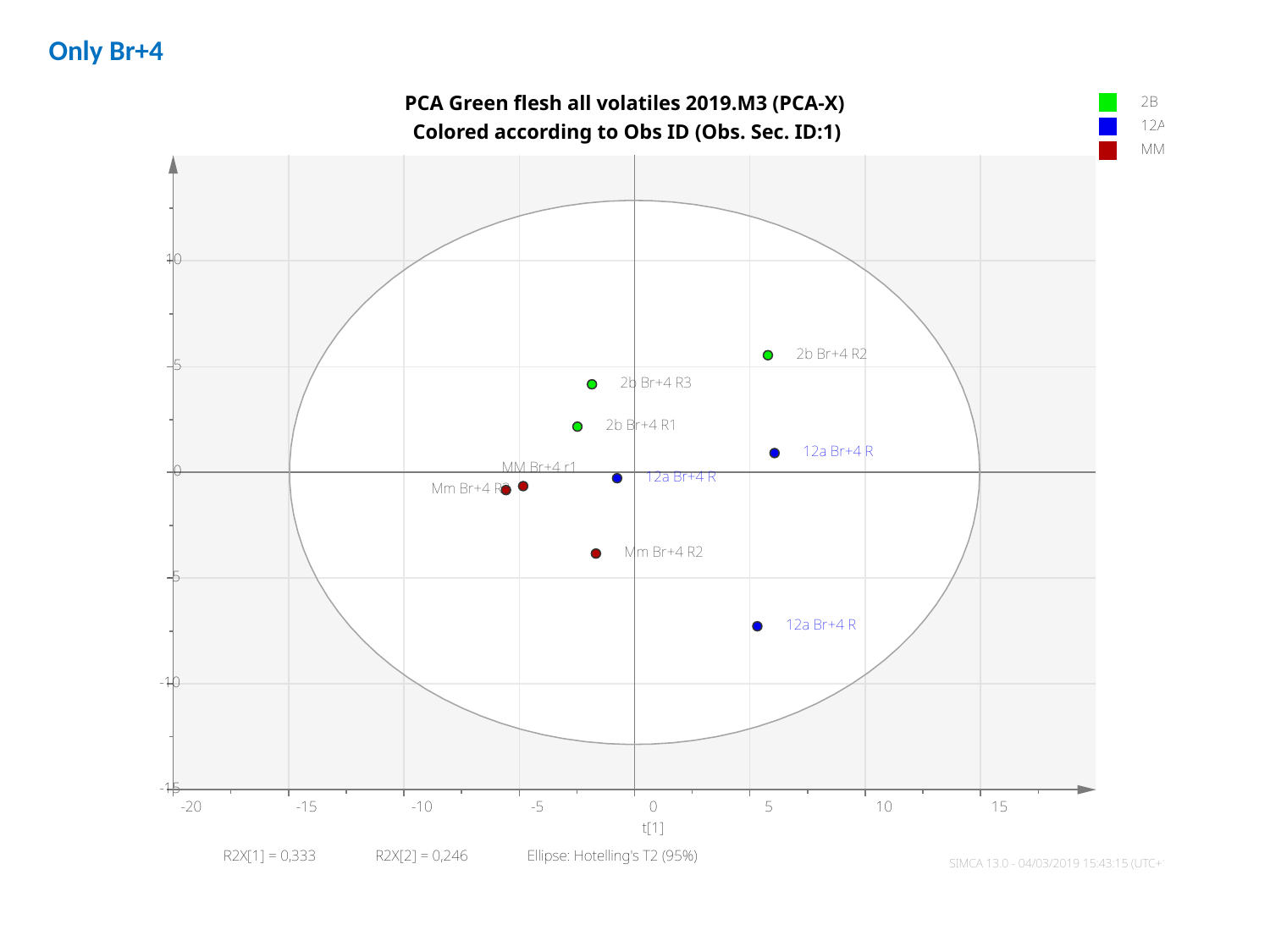

Only Br+4

## Slide 5
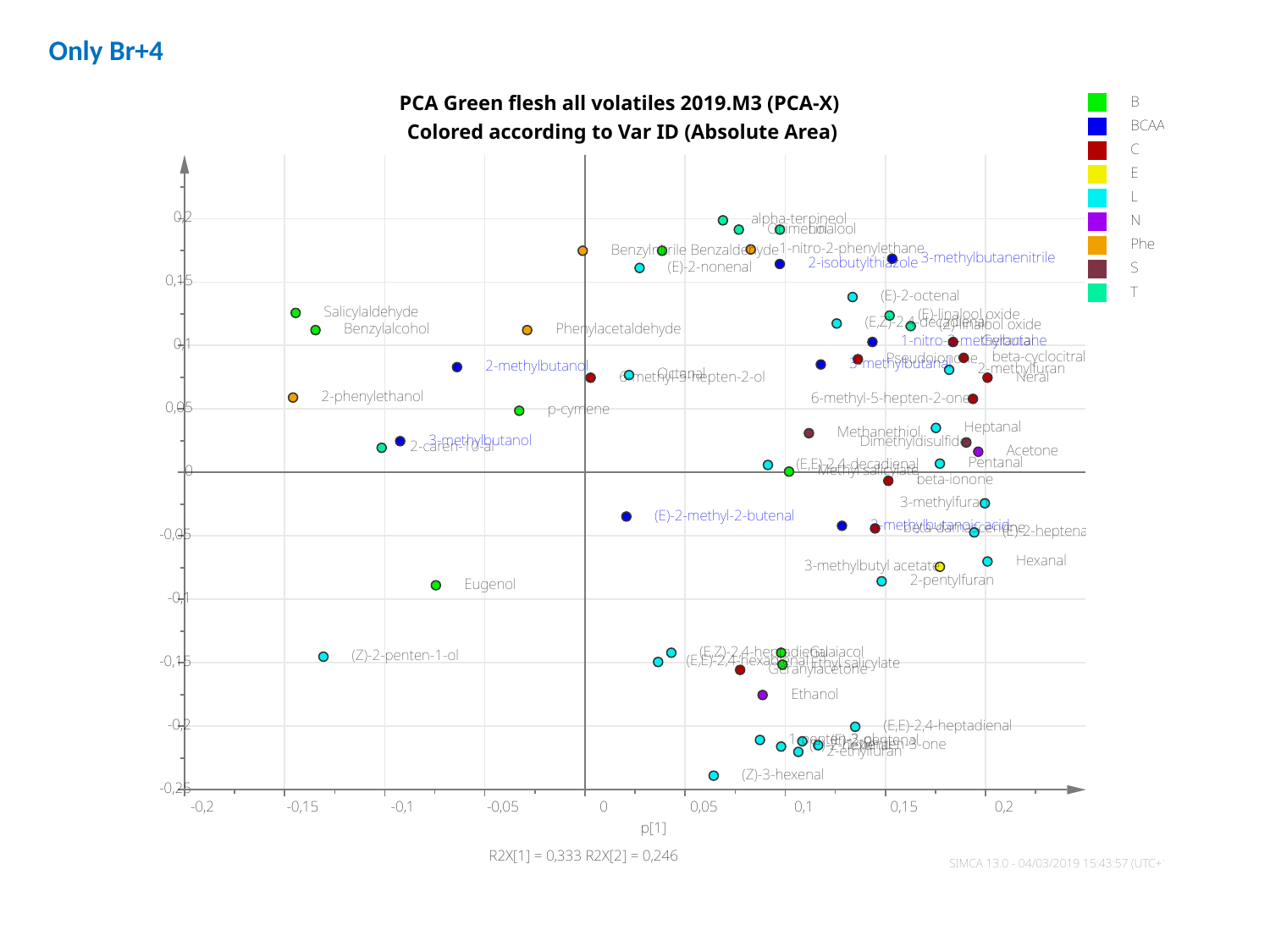

Only Br+4

## Slide 6
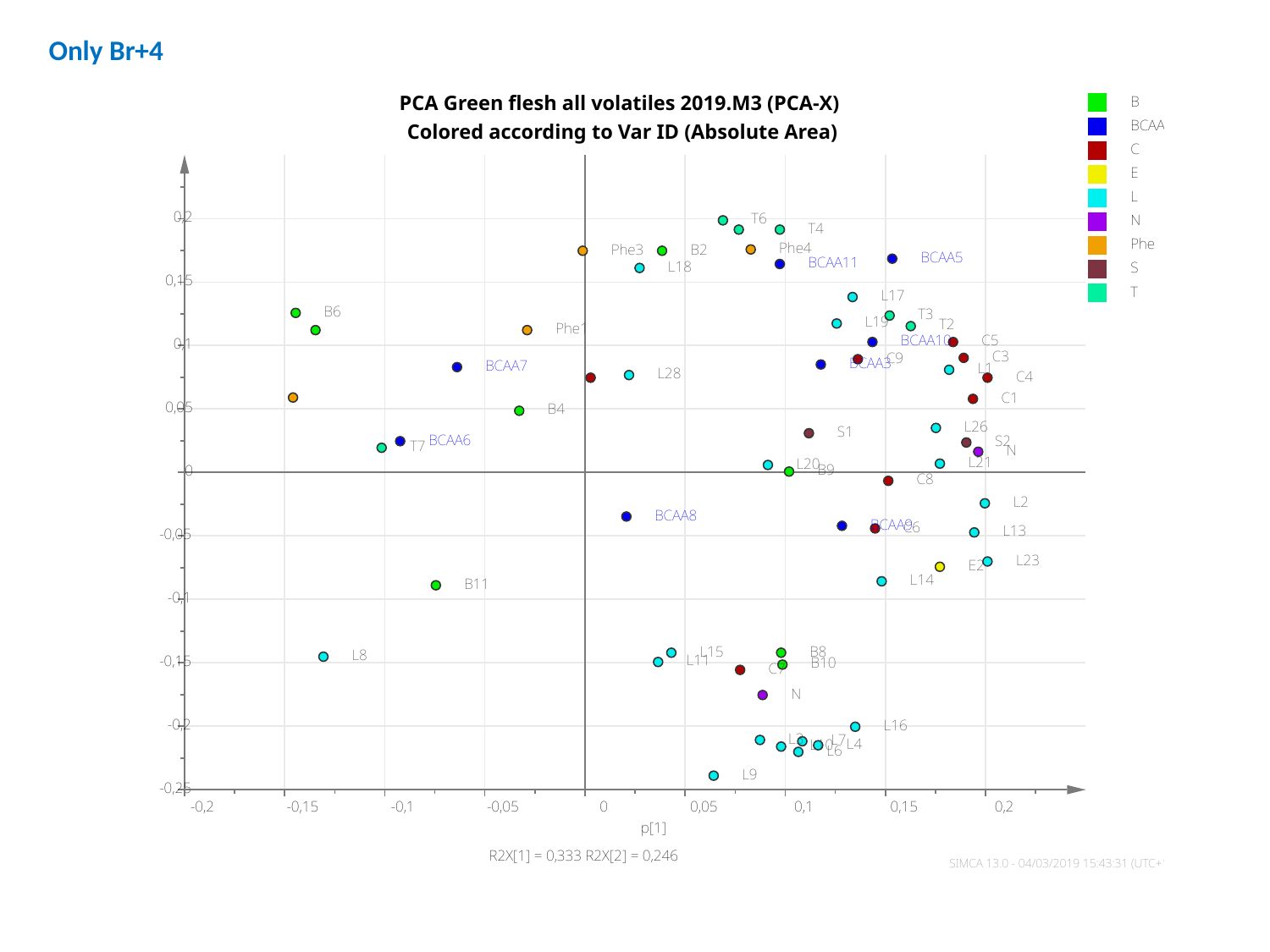

Only Br+4

Supplement: Supplementary file 6 [file Presentation_2.PPTX]

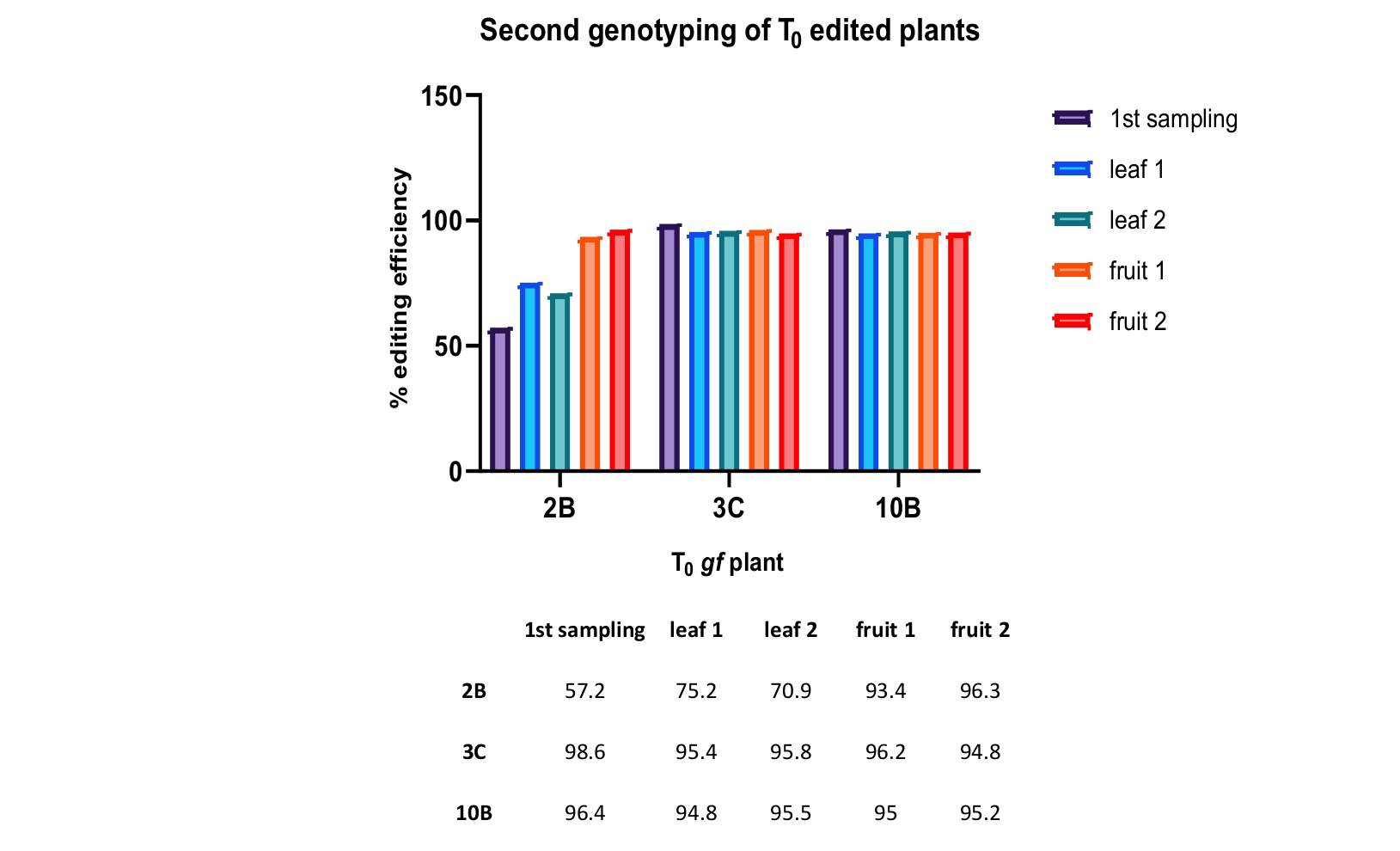

Supplement: Supplementary file 7 [file Image_1.TIF]

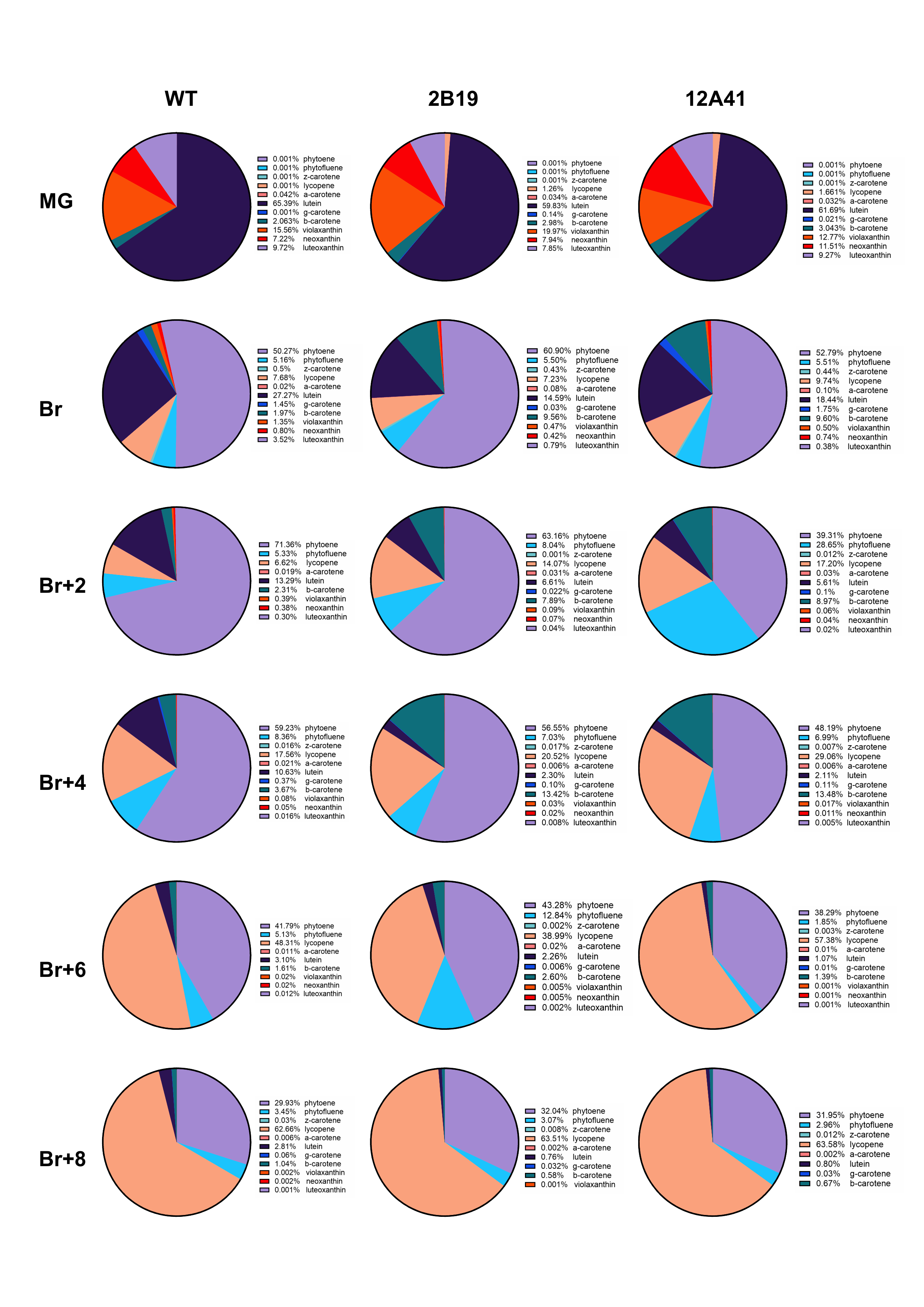

Supplement: Supplementary file 8 [file Image_2.TIF]

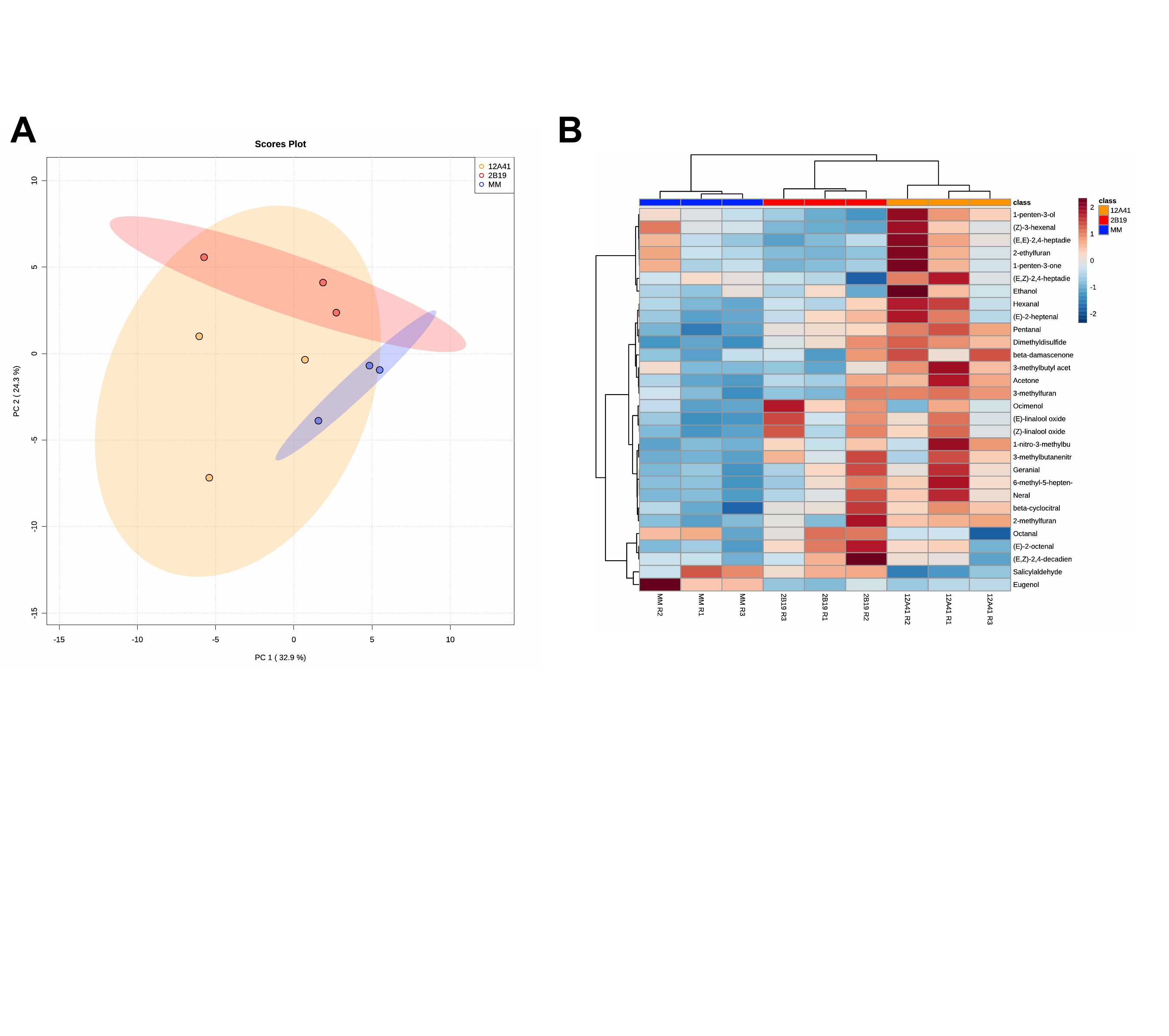

Supplement: Supplementary file 9 [file Image_3.TIF]

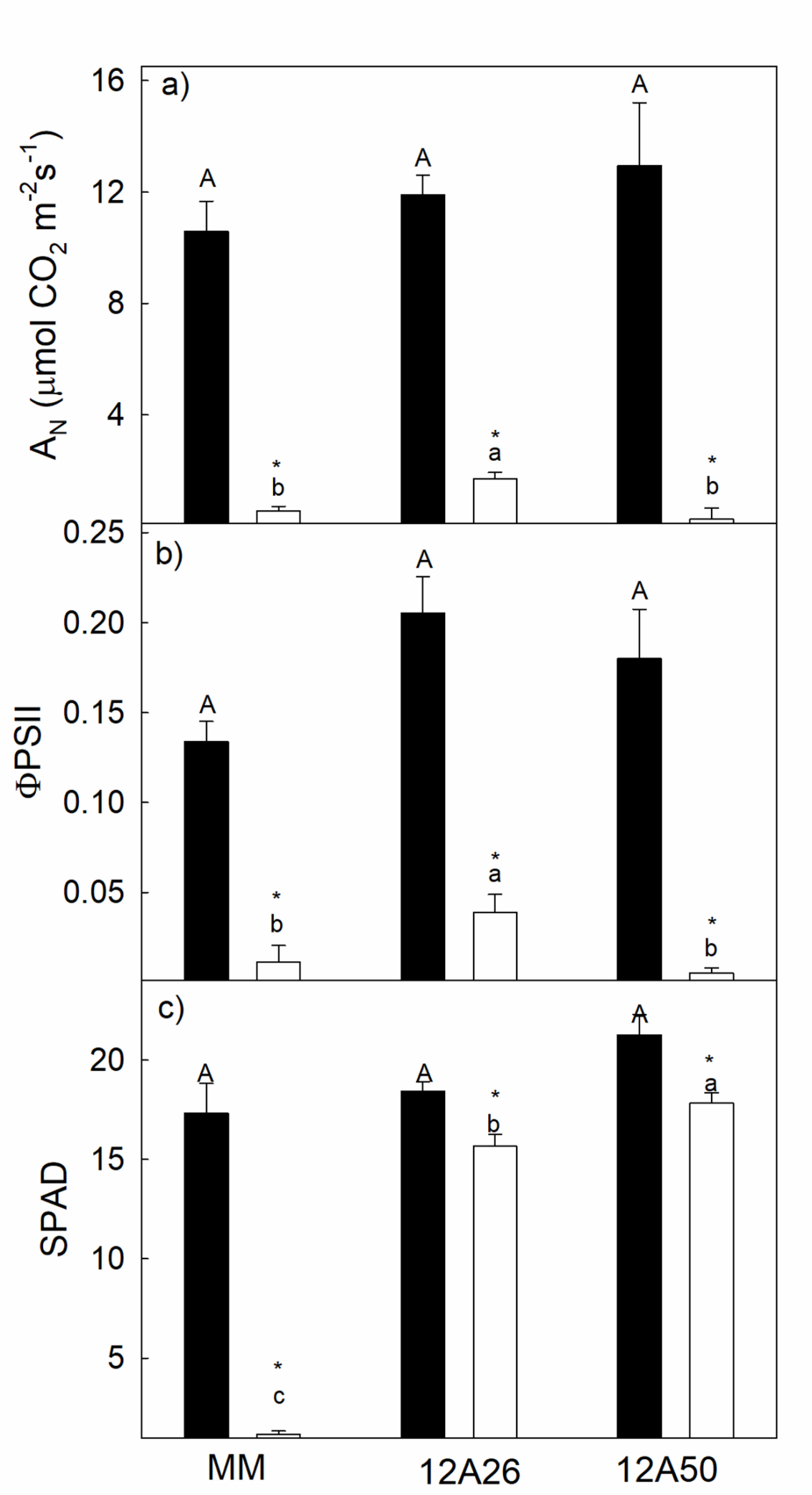

Supplement: Supplementary file 10 [file Image_4.TIF]
